# Modulation of the NOTCH1 Pathway by LUNATIC FRINGE Is Dominant over That of MANIC or RADICAL FRINGE

**DOI:** 10.3390/molecules26195942

**Published:** 2021-09-30

**Authors:** Florian Pennarubia, Alison V. Nairn, Megumi Takeuchi, Kelley W. Moremen, Robert S. Haltiwanger

**Affiliations:** Complex Carbohydrate Research Center, Department of Biochemistry and Molecular Biology, University of Georgia, Athens, GA 30602, USA; Florian.Pennarubia@uga.edu (F.P.); avnairn@ccrc.uga.edu (A.V.N.); megumit@uga.edu (M.T.); moremen@uga.edu (K.W.M.)

**Keywords:** NOTCH1, Fringe, EGF-like domains, *O*-fucosylation, glycosylation

## Abstract

Fringes are glycosyltransferases that transfer a GlcNAc to *O*-fucose residues on Epidermal Growth Factor-like (EGF) repeats. Three Fringes exist in mammals: LUNATIC FRINGE (LFNG), MANIC FRINGE (MFNG), and RADICAL FRINGE (RFNG). Fringe modification of *O*-fucose on EGF repeats in the NOTCH1 (N1) extracellular domain modulates the activation of N1 signaling. Not all *O*-fucose residues of N1 are modified by all Fringes; some are modified by one or two Fringes and others not modified at all. The distinct effects on N1 activity depend on which Fringe is expressed in a cell. However, little data is available on the effect that more than one Fringe has on the modification of *O*-fucose residues and the resulting downstream consequence on Notch activation. Using mass spectral glycoproteomic site mapping and cell-based N1 signaling assays, we compared the effect of co-expression of N1 with one or more Fringes on modification of *O*-fucose and activation of N1 in three cell lines. Individual expression of each Fringe with N1 in the three cell lines revealed differences in modulation of the Notch pathway dependent on the presence of endogenous Fringes. Despite these cell-based differences, co-expression of several Fringes with N1 demonstrated a dominant effect of LFNG over MFNG or RFNG. MFNG and RFNG appeared to be co-dominant but strongly dependent on the ligands used to activate N1 and on the endogenous expression of Fringes. These results show a hierarchy of Fringe activity and indicate that the effect of MFNG and/or RFNG could be small in the presence of LFNG.

## 1. Introduction

The Notch signaling pathway is essential for the regulation of developmental processes and for the maintenance of homeostasis in adult tissues [1]. This signaling pathway is initiated by interaction of Notch receptors with their canonical ligands Delta-like (DLL1 and 4) and JAGGED (JAG1 and 2) [2]. Four Notch receptors exist in mammals (NOTCH1-4). Their extracellular domains contain 29–36 Epidermal Growth Factor-like (EGF) repeats, each EGF consisting of 30 to 50 amino acids, characterized by the presence of 6 cysteines linked by 3 disulfide bonds (Cys1-Cys3, Cys2-Cys4, Cys5-Cys6) [3]. Many of the EGFs in Notch receptors are known to be modified by *O*-glycans [4,5,6,7,8], including *O*-fucose [4,7,9]. Protein *O*-fucosyltransferase 1 (POFUT1) [7,10] catalyzes the *O*-fucosylation of properly folded EGFs containing the consensus sequence C^2^XXXX(**S**/**T**)C^3^, where C^2^ and C^3^ are the second and third conserved cysteines in the EGF [11,12]. *O*-fucosylation of NOTCH1 (N1) is essential for its trafficking and function [13,14,15,16]. In particular, the *O*-fucose residues on N1 EGF repeat numbers 8 and 12 (EGF8 and EGF12) are directly involved in the interaction of the receptor with its ligands [17,18], and loss of these residues strongly affects Notch signaling [4].

*O*-fucose residues can be extended by a GlcNAc, then a galactose, and finally a sialic acid to form a tetrasaccharide [4,19]. The transfer of a GlcNAc onto an *O*-fucose is mediated by glycosyltransferases of the Fringe family comprising three members: LUNATIC FRINGE (LFNG), MANIC FRINGE (MFNG), and RADICAL FRINGE (RFNG) [4,20]. Among the three members of the Fringe family, LFNG is the most important for development, ensuring proper functioning of several critical Notch-dependent processes. Elimination of *Lfng* in mice results in severe defects in somitogenesis, an N1-dependent process [21,22], and homozygous inactivating mutations in LFNG cause similar defects in humans [23]. In contrast, MFNG and RFNG are not necessary for normal embryonic development [24,25,26,27]. Cell-based signaling assays demonstrated that co-expression of LFNG or MFNG with N1 promotes the induction of N1 activation by DLL1 but inhibits activation by JAG1 while RFNG promotes both [4]. It was proposed that this is due to the specificity of these three Fringes to modify *O*-fucose residues on certain EGFs. LFNG, MFNG, and RFNG all modified the *O*-fucose carried by EGF8, and the presence of an elongated *O*-fucosylglycan at this position promoted N1 activation by DLL1 [4]. Fringe modification of *O*-fucose on EGF6 and 36 inhibited the signal induced by JAG1. Unlike LFNG or MFNG, RFNG activity toward *O*-fucose on EGF6 and/or 36 was extremely low to absent, explaining why RFNG expression did not inhibit the activation of the Notch pathway by JAG1 [4]. Elongation of *O*-fucose on EGF12 by LFNG or RFNG, but not by MFNG, promoted N1 activation by both DLL1 and JAG1. Consequently, the action of RFNG on EGF12 promotes activation of the Notch pathway by JAG1 [4]. Thus, modification of specific *O*-fucose residues of N1 by Fringes plays a key role in modulation of N1 activity [13,28].

Several studies have shown that LFNG, MFNG, and/or RFNG can be expressed within the same cell. This is notably the case for T cell progenitors and naive CD4^+^T cells where the three Fringes are expressed and associated with T cell differentiation [29,30]. The presence of mRNA encoding two or three members of the Fringe family has also been reported in other cell types, suggesting the simultaneous presence of several Fringes in retinal endothelial cells [31], basal cells of the skin [32], granulosa [26], and Sertoli and germ cells [33]. However, the effect of simultaneous expression of more than one Fringe in a cell on N1 receptor *O*-fucose modification and consequently on pathway activation has been poorly explored. One report evaluating T cell progenitors expressing none, one, or all three Fringes showed that all three contribute to optimal Notch pathway activation, although the effect of LFNG appears to be more significant than MFNG or RFNG alone [29].

In this work, we used a cell-based signaling assay to compare the ability of DLL1 or JAG1 to activate N1 co-expressed with or without a single Fringe in three different cell lines extensively used for N1 signaling assays: U2OS, NIH3T3, and CHO [4,8,34,35,36,37,38,39]. Mass spectral glycoproteomic analysis of a secreted portion of the N1 extracellular domain co-expressed with or without each Fringe separately was performed to analyze which EGFs were modified. These experiments were then repeated to analyze the effect of the presence of two or three Fringes on the glycan profile of N1 and on the activation of its associated pathway. Our results revealed a dominant effect of LNFG over MFNG and RFNG both in the glycan profile of the receptor and in the effects on activation of N1 by DLL1 and JAG1. The dominance of MFNG over RFNG varies when JAG1 is the activated ligand. Altogether, these data suggest a hierarchy of the Fringe effect with a dominance of LFNG over MFNG and RFNG.

## 2. Results

### 2.1. The Modification of NOTCH1 O-Fucose Sites by Endogenous Fringes in Different Cells Determines the Efficiency of Its Stimulation by Ligands

**Figure 1:**
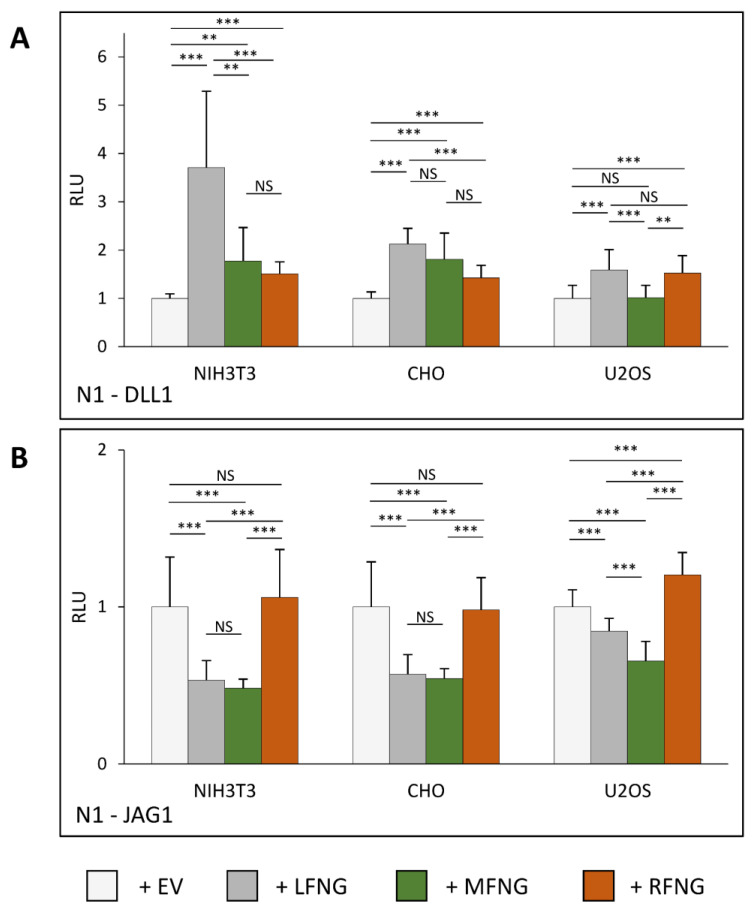
Effects of individual Fringes on N1 activation in NIH3T3, CHO, and U2OS cell lines. NIH3T3, CHO, and U2OS were co-transfected with plasmids encoding full-length mouse NOTCH1 (N1) and empty vector (EV), LFNG, MFNG, or RFNG, then co-cultured with L-cells stably overexpressing (**A**) DLL1 or (**B**) JAG1. Relative Luciferase units (RLU) compared to controls (EV) (normalized to 1 for each ligand and cell line) were determined. Statistical significance was calculated using one-way ANOVA. Bar graph shows mean +/− SD; four or five independent experiments (depending on the cell line) n = 8 to 10 were analyzed (*** *p* < 0.005; ** *p* < 0.01; NS: no significance).

Our first objective was to determine how co-expression of N1 with members of the Fringe family (LFNG, MFNG, or RFNG) modulates Notch signaling within three cell lines commonly used for Notch activation assays (NIH3T3, CHO, and U2OS). To this purpose, mouse N1 was expressed in these cell lines in the presence or absence of co-expressed mouse LFNG, MFNG, or RFNG. The transfected cells were then co-cultured with L cells stably expressing DLL1 or JAG1 (Figure 1A,B). The NIH3T3 and CHO cells showed a similar profile to previously published results [4,8] for induction of N1 signaling by DLL1. All three Fringes enhanced N1 activation by DLL1, but LFNG induced the strongest enhancement of signal compared to MFNG or RFNG. Interestingly, N1 activation in NIH3T3 cells was more sensitive to the presence of LFNG than in CHO cells. Activation of N1 by DLL1 was different in U2OS cells compared to NIH3T3 and CHO cells: LFNG overexpression had less of an effect and MFNG had no effect on N1 signaling (Figure 1A). The results obtained with the activation of the N1 pathway by JAG1 are similar between the three cell lines. Namely, a decrease in N1 signal induction by JAG1 with the co-expression of LFNG or MFNG, although the effect of LFNG was lower in the case of U2OS cells. RFNG tended to increase the signal (significant only for U2OS). However, we did not observe a strong increase in N1 signaling in the presence of RFNG in NIH3T3 cells as described in previous studies [4] (Figure 1B).

**Figure 2:**
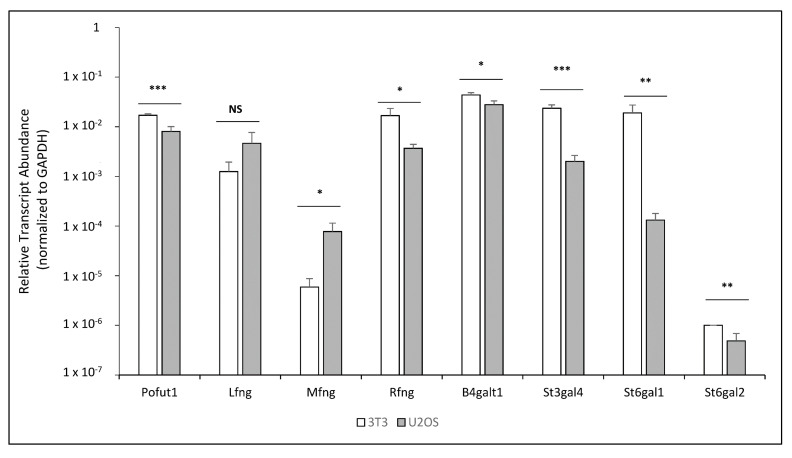
NIH3T3 and U2OS comparison by qRT-PCR of relative mRNA levels for endogenous glycosyltransferases involved in the *O*-fucosylation of EGF repeats. The relative transcript levels of all glycosyltransferases involved in *O*-fucosylation were analyzed. Relative quantification, normalized with GAPDH, for each gene was determined and plotted on a log_10_ scale. Statistical significance was calculated using one-way ANOVA. Bar graph shows mean +/− SD; three (NIH3T3) or four (U2OS) independent experiments (*** *p* < 0.005; ** *p* < 0.01; * *p* < 0.05; NS: no significance).

To help explain these results, a qRT-PCR analysis was performed to analyze the relative levels of mRNA for endogenous glycosyltransferases involved in the transfer of *O*-fucose glycans and their elongation in the NIH3T3 and U2OS lines. The CHO cell line was excluded from the analysis due to the absence of suitable primers in our laboratory; however, the results obtained by cell-based N1 co-culture signaling assays were similar to those of the NIH3T3, which can be used as a reference for this comparison. Interestingly, the relative transcript level of *Pofut1* was slightly lower in U2OS compared to NIH3T3 (Figure 2). However, as described below, this did not result in a change in the *O*-fucosylation of N1 peptides (Figure S1). The relative transcript level of *Lfng* was not significantly different between the two lines, but slightly higher levels of *Lfng* were present in U2OS. The level of *Mfng* was much higher in U2OS cells compared to NIH3T3, suggesting a potential explanation for the lack of MFNG effects on N1 activation by DLL1 in U2OS cells (Figure 1A). The level of *Rfng* is lower in U2OS (Figure 2). The relative levels of glycosyltransferases involved in elongation of *O*-fucose beyond the disaccharide (*B4galt1*, *St3gal4*, *St6gal1*, *St6gal2*) were also lower in U2OS (Figure 2).

**Figure 3:**
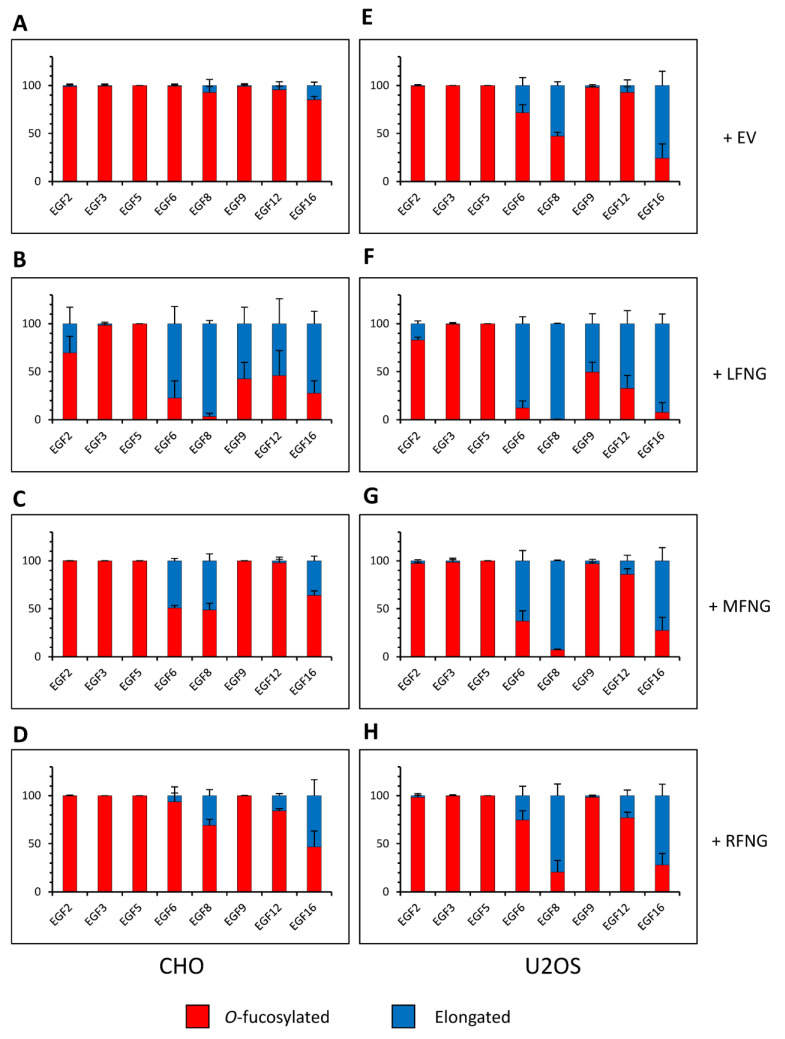
Site-specific *O*-fucose elongation on N1 EGF1-18 expressed in CHO and U2OS cell lines. Quantification of the percentage of peptides modified by a monosaccharide *O*-fucose (red) and elongated *O*-fucose (blue) by at least a GlcNAc. Extracted ion chromatograms (EICs) of the mass spectrometry (MS) data of peptides derived from mouse N1 EGF1-18 overexpressed in (**A**–**D**) CHO and (**E**–**H**) U2OS were used for the quantification. (**A**,**E**) Endogenous elongation of *O*-fucoses of N1. (**B**,**F**) Elongation of *O*-fucoses of N1 in the presence of LFNG. (**C**,**G**) Elongation of *O*-fucoses of N1 in the presence of MFNG. (**D**,**H**) Elongation of *O*-fucoses of N1 in the presence of RFNG. The data used to generate the EICs are available in Tables S4–S15 and S28–S39. The ions used to generate the EICs are in Table S3. Bar graph shows mean +/− SD; three or four independent experiments (depending on the cell line) n = 3 to 4 were analyzed.

In order to analyze the *O*-fucosylation and elongation profile of N1 within our three cell lines and to try to correlate it with the previously obtained results, mass spectral glycoproteomic site analysis was performed. A truncated form of the N1 extracellular domain containing EGFs 1-18 was produced in a secreted form, purified from the medium, and analyzed. N1 EGF1-18 contains the most significant regions necessary for ligand binding, and longer forms of extracellular domain are difficult to produce in the amounts needed to perform this analysis. We first analyzed the *O*-fucose glycans on N1 EGF1-18 expressed in NIH3T3, CHO, and U2OS cells in the absence of any exogenous Fringe (Figure S1). The NIH3T3 (Figure S1A,D) and CHO (Figure 3A and Figure S1B,E) cell samples had a similar profile with very little elongated *O*-fucose. Unfortunately, the expression of N1 EGF1-18 in NIH3T3 cells was too low to allow us to analyze or to consistently replicate all the *O*-fucosylation sites (Figure S1A,D). In all cases, all EGFs containing POFUT1 consensus sequences were modified at high stoichiometry with *O*-fucose monosaccharide. In CHO cells, only *O*-fucose glycans on EGF8, 12, and 16 were slightly (<15%) elongated past the monosaccharide. Considering the total amount of elongated *O*-fucose (di-, tri-, and tetrasaccharide), the percentage of elongated *O*-fucose on EGF8, 12, and 16 in CHO cells was 7.3% (±3.6), 4.5% (±1.0), and 14.9% (±2.0), respectively (Figure 3A and Figure S1E). In contrast, N1 *O*-fucose residues were elongated more extensively in the U2OS cells. Elongated forms of *O*-fucose on EGF8, 12, and 16 were 52.8% (±2.3), 7.3% (±3.0), and 75.7% (± 8.5), respectively (Figure 3E and Figure S1F). In addition, *O*-fucose carried by EGF6 was elongated to 28.3% (±4.2) in U2OS cells, whereas it was a monosaccharide in both NIH3T3 (Figure S1D) and CHO cells (Figure 3A and Figure S1E).

Overexpression of LFNG, MFNG, and RFNG resulted in modification of *O*-fucose on specific EGFs in CHO and U2OS cells (Figure 3). LFNG modified the most EGFs: EGF2, 6, 8, 9, 12, and 16 (Figure 3B,F). MFNG only modified *O*-fucose carried by EGF6, 8, and 16 (Figure 3C,G) and RFNG those carried by EGF8, 12, and 16 (Figure 3D,H). We also saw Fringe elongation at EGF18, but due to its position at the C-terminus of the protein, which frees it from the conformational constraints present for the whole receptor containing 36 EGF repeats, and the fact that we could not reliably detect the *O*-fucosylated peptide from EGF18 (Figure S3), we did not include EGF18 in our analysis. The proportions of the different *O*-fucose glycoforms also appear to be specific to each cell line (Figures S1 and S2).

### 2.2. The Effect of LFNG on N1 Signaling Is Dominant over MFNG and RFNG

As seen previously [4], the independent overexpression of each Fringe induces a specific effect on N1 activation. However, the presence of two or three of these enzymes within the same cell [26,29,30,31,32,33] suggests the possibility of simultaneous action. This could lead to complementarity or, on the contrary, to a process of competition or even inhibition. To analyze the effect of the simultaneous presence of multiple Fringes within the same cell, different co-overexpression conditions were performed using the same total amount of transfected DNA. Thus, N1 was co-expressed with empty vector (EV), LFNG, MFNG, RFNG, LFNG + MFNG, LFNG + RFNG, MFNG + RFNG, and LFNG + MFNG + RFNG in each of our three cell lines.

**Figure 4:**
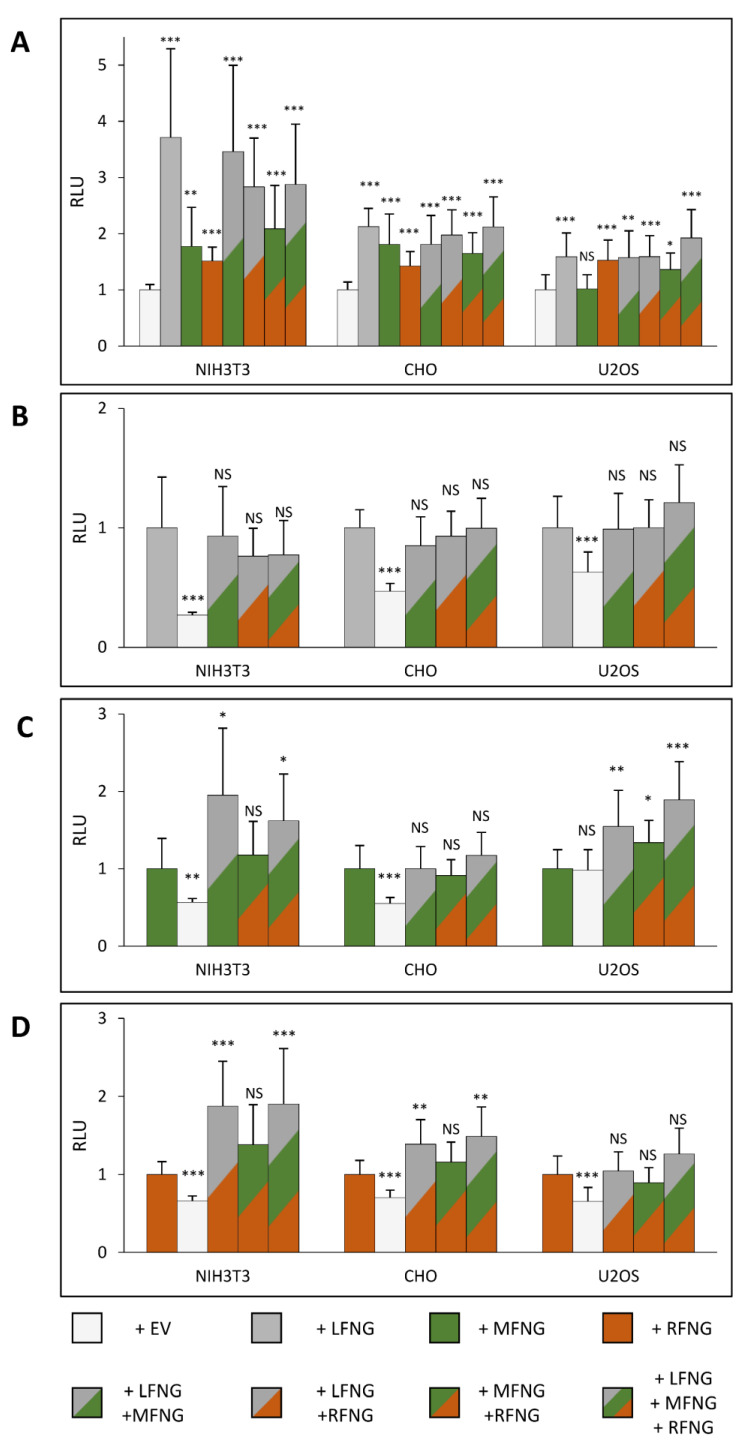
Effect of expressing multiple Fringes on NOTCH1 activation by DLL1. NIH3T3, CHO, and U2OS were co-transfected with plasmids encoding full-length mouse NOTCH1 (N1) and empty vector (EV) or LFNG and/or MFNG and/or RFNG. Then, cell-based co-culture N1 activation assays were performed with L cells stably overexpressing DLL1 as in Figure 1A. Relative Luciferase units (RLU) normalized to EV controls (**A**), to LFNG (**B**), to MFNG (**C**), or to RFNG, (**D**) (normalized to 1 for each ligand and cell line) were determined. Statistical significance was calculated using one-way ANOVA. Bar graph shows mean +/− SD; four or five independent experiments (depending on the cell line) n = 8 to 10 were analyzed (*** *p* < 0.005; ** *p* < 0.01; * *p* < 0.05; NS: no significance).

In the case of the induction of N1 signaling by DLL1, the presence of multiple Fringes induced an overall increase in signal intensity in three cell lines (Figure 4A). The overexpression of LFNG with MFNG and/or RFNG, induced a signal similar to the overexpression of LFNG alone, in all three cell types (Figure 4A). Indeed, the comparison of LFNG alone with LFNG + MFNG, LFNG + RFNG, and LFNG + MFNG + RFNG revealed no significant difference (Figure 4B). Consistent with these results, comparison of MFNG alone with LFNG + MFNG and LFNG + MFNG + RFNG, as well as RFNG alone with LFNG + RFNG and LFNG + MFNG + RFNG showed that the presence of LFNG increased the signal significantly (Figure 4C,D). However, two exceptions were observed: no significant difference in CHO cells between MFNG only and MFNG + LFNG (Figure 4C), and in U2OS between RFNG only and RFNG + LFNG (Figure 4D). This is probably explained by the fact that there is no significant difference between the effects of expression of LFNG or MFNG in CHO cells and between LFNG or RFNG in U2OS (Figure 1A). Regarding the simultaneous overexpression of MFNG + RFNG, it seems to induce a signal similar to MFNG or RFNG alone in the NIH3T3 and CHO cells (Figure 4C,D). In addition, RFNG is slightly dominant over MFNG in U2OS cells (Figure 4C), likely due to RFNG’s ability to modify EGF12 more efficiently than MFNG (Figure 3G,H).

Our prior data suggests that Fringe modulates N1 signaling induced by DLL1 by the elongation of *O*-fucose on EGF8 and 12 [4]. Single mutants of the *O*-fucosylation sites of EGFs 8 (T^311^V) and 12 (T^466^V) were used to test whether LFNG dominance could be associated with these two essential sites. Interestingly, the mutant T^466^V suppressed the dominance of LFNG over MFNG previously seen in NIH3T3 and U2OS cells (Figure S4A,C) and eliminated the dominance of LFNG over RFNG in CHO cells but not in NIH3T3 (Figure S4D,E). This mutant also eliminated the dominance of RFNG over MFNG in U2OS (Figure S4C).

The mutant T^311^V suppressed the dominance of LFNG over MFNG and RFNG (Figure S4A,D,E), except in U2OS, where LFNG retained its dominant effect on MFNG (Figure S4C). This could be due to the strong endogenous modification of EGF8 in U2OS cells and thus the inability of LFNG to exert its dominance by modifying this EGF more strongly than MFNG (Figure 3F,G).

**Figure 5:**
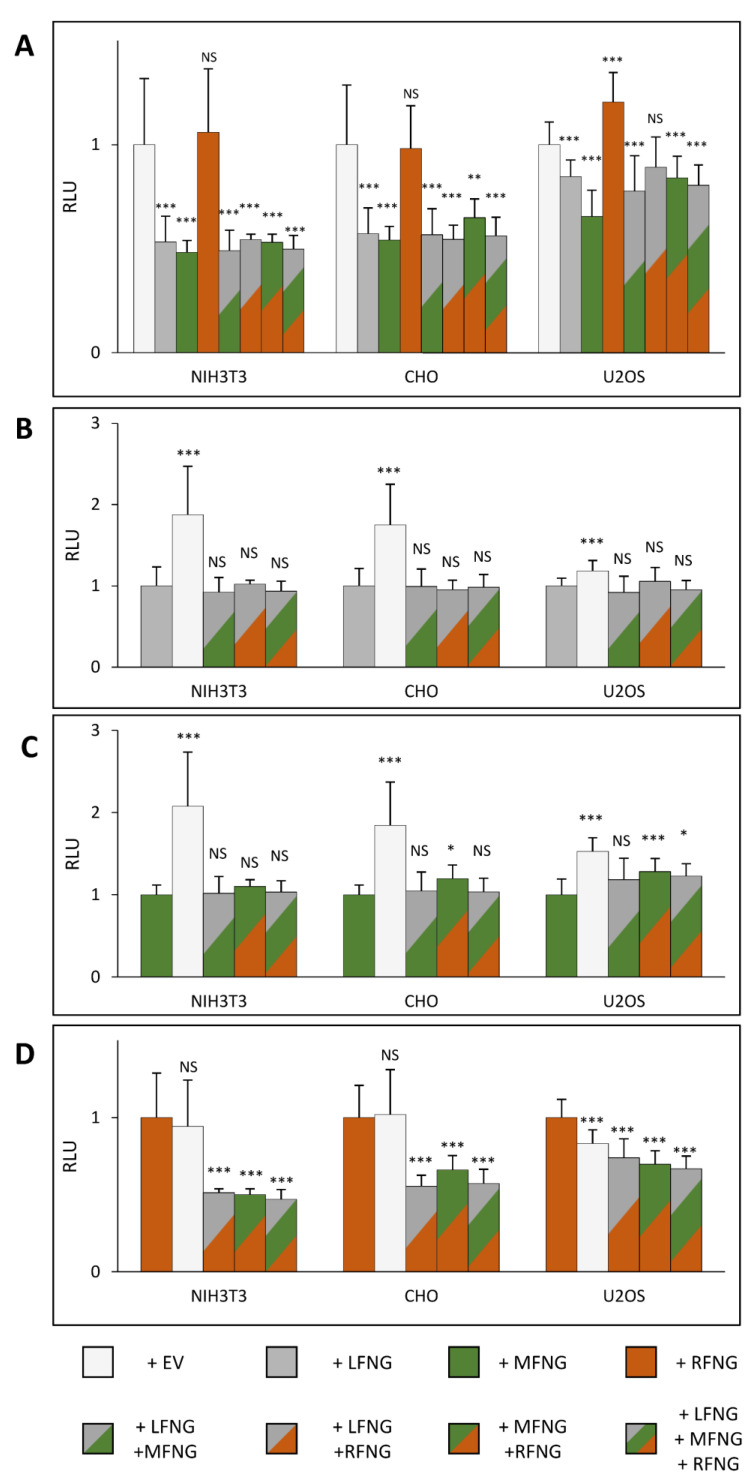
Effect of expressing multiple Fringes on NOTCH1 pathway activation by JAG1. NIH3T3, CHO, and U2OS were co-transfected with plasmids encoding full-length mouse NOTCH1 (N1) and empty vector (EV) or LFNG and/or MFNG and/or RFNG. Then, cell-based co-culture N1 activation assays were performed with L cells stably overexpressing JAG1 as in Figure 1B. Relative Luciferase units (RLU) normalized to EV controls (**A**), to LFNG (**B**), to MFNG (**C**)**,** or to RFNG, (**D**) (normalized to 1 for each ligand and cell line) were determined. Statistical significance was calculated using one-way ANOVA. Bar graph shows mean +/− SD; four or five independent experiments (depending on the cell line) n = 8 to 10 were analyzed (*** *p* < 0.005; ** *p* < 0.01; * *p* < 0.05; NS: no significance).

For the induction of N1 signaling by JAG1, the presence of LFNG or MFNG inhibited signaling in all three cell lines, while RFNG caused no decrease or a slight increase (Figure 5A). Co-expression of LFNG and MFNG did not cause any further decrease in N1 activation by JAG1 (Figure 5B,C). In contrast, co-expression or RFNG with either LFNG or MFNG caused a significant decrease in N1 activation by JAG1, suggesting that both LFNG and MFNG are dominant over RFNG for inhibition of JAG1-N1 activity (Figure 5D). Interestingly, MFNG + RFNG induced slightly less inhibition than MFNG only in CHO and U2OS cells (Figure 5C).

We have previously shown that Fringe elongation of the *O*-fucose carried by EGF6 and EGF36 inhibited the activation of the N1 receptor by JAG1 [4]. Thus, the mutants at the *O*-fucosylation sites of EGF6 (T^232^V) and 36 (T^1402^V) were used to understand these dominance mechanisms. The T^232^V mutation resulted in loss of dominance of LFNG and MFNG over RFNG (Figure S5). However, mutant T^1402^V induced a loss of dominance only in NIH3T3, highlighting a weaker effect of EGF36 *O*-fucose elongation compared to EGF6.

### 2.3. The Dominance of LFNG over MFNG and RFNG Is Due to the Ability to Modify a Larger Number of O-Fucose Sites with Greater Efficiency

**Figure 6:**
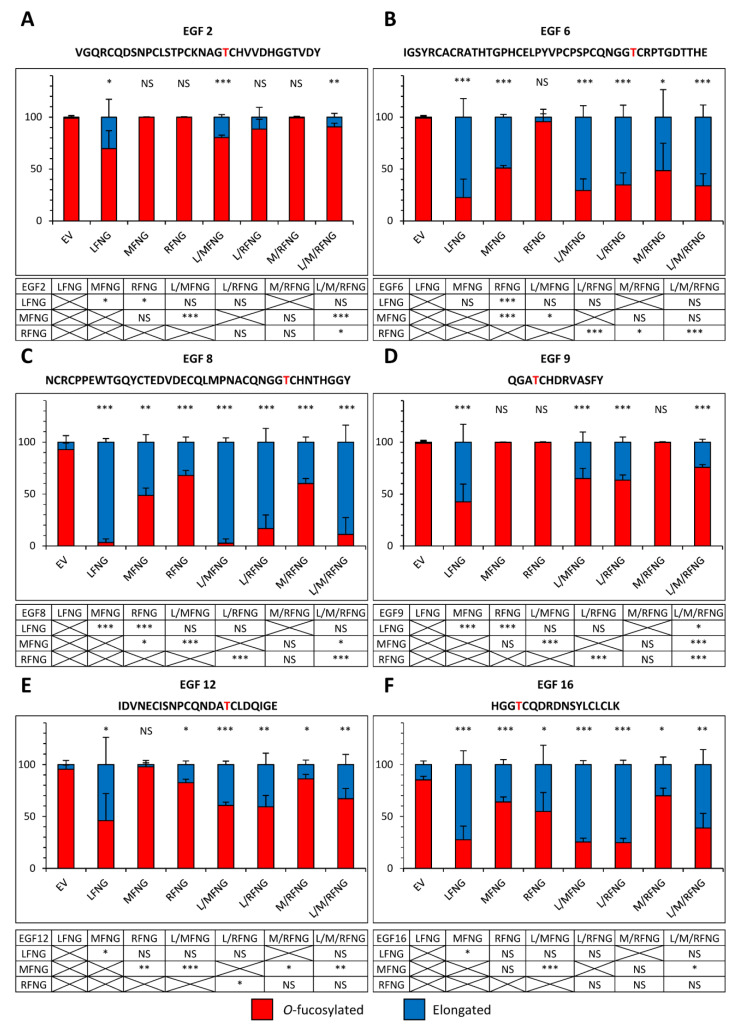
Site-specific *O*-fucosylation profile of N1 EGF1-18 co-expressed with multiple Fringes in CHO cells. Quantification of the percentage of peptides modified by a monosaccharide *O*-fucose (red) and elongated *O*-fucose (blue) by at least a GlcNAc. Extracted ion chromatograms (EICs) of the mass spectrometry (MS) data of peptides for EGF2 (**A**), 6 (**B**), 8 (**C**), 9 (**D**), 12 (**E**) and 16 (**F**) derived from mouse N1 EGF1-18 overexpressed with EV, LFNG, MFNG, RFNG, LFNG + MFNG, LFNG + RFNG, MFNG + RFNG, or LFNG + MFNG + RFNG in CHO were used for the quantification. Statistical significance was calculated using one-way ANOVA. Bar graph shows mean +/− SD; three independent experiments n = 3 were analyzed (*** *p* < 0.005; ** *p* < 0.01; * *p* < 0.05; NS: no significance). The data used to generate the EICs are available in Tables S4–S27. The ions used to generate the EICs are in Table S3.

**Figure 7:**
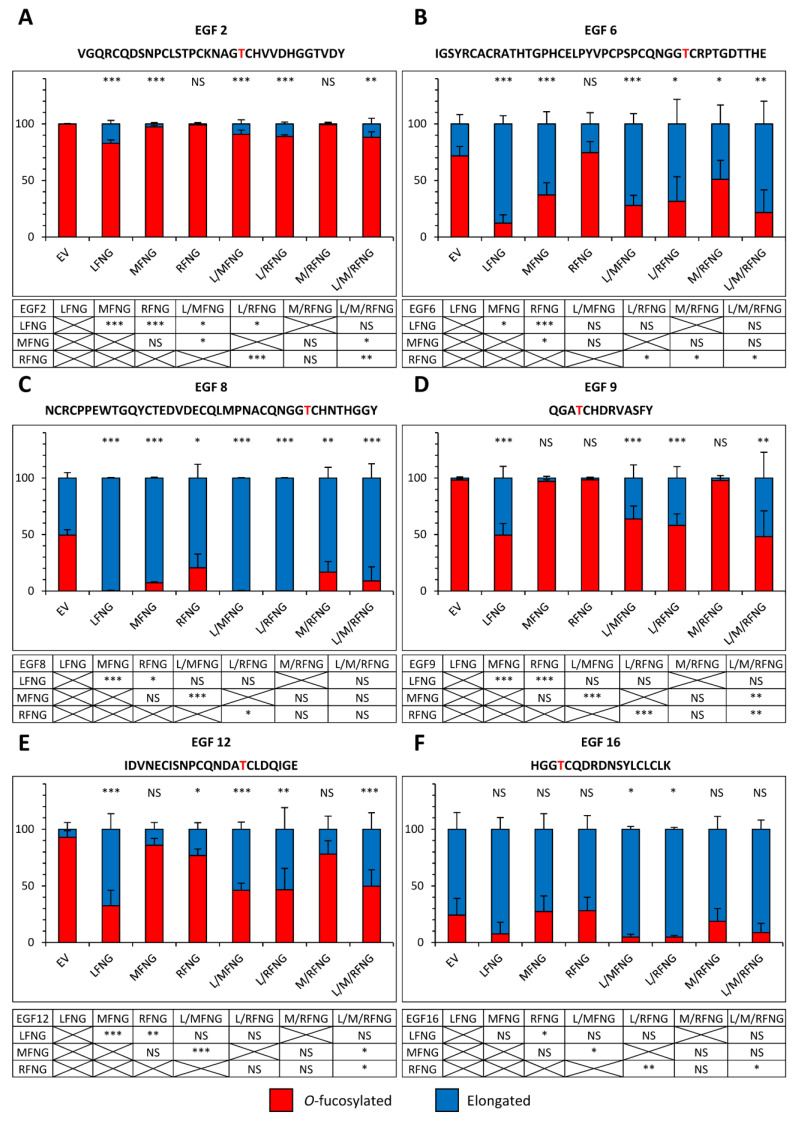
Site-specific *O*-fucosylation profile of N1 EGF1-18 co-expressed with multiple Fringes in U2OS cells. Quantification of the percentage of peptides modified by a monosaccharide *O*-fucose (red) and elongated *O*-fucose (blue) by at least a GlcNAc. Extracted ion chromatograms (EICs) of the mass spectrometry (MS) data of peptides for EGF2 (**A**), 6 (**B**), 8 (**C**), 9 (**D**), 12 (**E**) and 16 (**F**) derived from mouse N1 EGF1-18 overexpressed with EV, LFNG, MFNG, RFNG, LFNG + MFNG, LFNG + RFNG, MFNG + RFNG, or LFNG + MFNG + RFNG in U2OS were used for the quantification. Statistical significance was calculated using one-way ANOVA. Bar graph shows mean +/− SD; four or five independent experiments n = 3 to 4 were analyzed (*** *p* < 0.005; ** *p* < 0.01; * *p* < 0.05; NS: no significance). The data used to generate the EICs are available in Tables S28–S51. The ions used to generate the EICs are in Table S3.

To better understand the results observed in the cell-based N1 co-culture signaling assays, glycoproteomic mass spectral analyses of secreted fragments of the N1 receptor including EGF1-18 in the presence of multiple Fringes (LFNG + MFNG, LFNG + RFNG, and LFNG + MFNG + RFNG) were performed (Figure 6 and Figure 7). As expected, *O*-fucose residues on EGF3 and 5 were not modified by any combination of Fringes (Figure S6). All other *O*-fucose residues carried by N1 EGF1-18 could be at least partially elongated by LFNG. Moreover, the action of LFNG was typically more efficient than MFNG or RFNG and the general pattern was that no combination of Fringe enzymes produced a significantly greater fraction of elongation than LFNG alone (Figure 6 and Figure 7).

When LFNG was combined with MFNG or RFNG or both, the elongated fraction was equivalent to or slightly lower (due to the lower proportion of *Lfng* DNA used for transfection) than that observed for LFNG alone. The combination of Fringes also produced a larger elongated fraction than that observed for MFNG or RFNG alone when their effect was lower than LFNG only (Figure 6 and Figure 7). As an example, the elongation of *O*-fucose on EGF9 in the presence of the EV was 1.0% (±1.0) in CHO and 1.9% (±0.4) in U2OS. In the presence of LFNG, MFNG, or RFNG only, this elongation was, respectively, 57.5% (±9.9), 0.2% (±0.1), and 0.3% (±0.2) in CHO and 50.5% (±5.2), 3.1% (±0.7), and 1.7% (±0.3) in U2OS. The combination of Fringes including LFNG (LFNG +MFNG, LFNG + RFNG, and LFNG + MFNG + RFNG) resulted in a respective elongation of 35.2% (±5.7), 36.6% (±2.8), and 24.3% (±1.6) in CHO and 36.3% (±6.7), 41.9% (±5.8), and 51.9% (±13.1) in U2OS, similar to LFNG only (Figure 6D and Figure 7D). Interestingly, the elongation of the *O*-fucose on EGF9 obtained with the co-overexpression of MFNG and RFNG was 0.3% (±0.3) in CHO and 2.4% (±1.2) in U2OS, similar to that observed in the presence of MFNG or RFNG alone (Figure 6D and Figure 7D). This suggests the absence of combined action to modify *O*-fucose on EGF9 and further supports the dominance of LFNG. LFNG dominance was also observed when all three Fringes were able to target the same *O*-fucose as in the case of the one carried by EGF8 (Figure 6C and Figure 7C). With EV, the elongation of this *O*-fucose was 7.3% (±3.6) in CHO and 50.5% (±2.8) in U2OS. The addition of LFNG, MFNG, or RFNG increased elongation, respectively, to 96.7% (±2.0), 51.4% (±4.1), and 32.2% (±2.9) in CHO and 99.8% (±0.2), 92.7% (±0.4), and 79.4% (±7.0) in U2OS. The combination of LFNG with MFNG and/or RFNG led to an elongation level similar to that observed for LFNG alone, i.e., 97.5% (±2.4), 83.3% (±7.6), and 88.9% (±9.4) in CHO and 99.8% (±0.2), 99.9% (±0.1), and 91.1% (±8.9) in U2OS for LFNG +MFNG, LFNG + RFNG, and LFNG + MFNG + RFNG, respectively. On the other hand, the combination of MFNG and RFNG resulted in an intermediate elongation between MFNG and RFNG (40.0% (±2.8) in CHO and 83.3% (±5.4) in U2OS) (Figure 6C and Figure 7C). Interestingly, when MFNG caused elongation but not RFNG, the MFNG + RFNG combination resulted in an elongation similar to MFNG. This was especially the case for their action on *O*-fucose carried by EGF6, where EV, MFNG, and RFNG were associated with an elongation of 0.9% (±0.9), 49.1% (±1.5), and 4.3% (±4.3) in CHO and 28.3% (±4.2), 62.8% (±6.2), and 25.5% (±5.7) in U2OS, respectively. The MFNG + RFNG combination resulted in an elongation of *O*-fucose on EGF6 of 51.6% (±15.3) in CHO and 49.1% (±11.8) in U2OS, showing dominance of MFNG over RFNG at EGF8 (Figure 6B and Figure 7B). The reverse was true considering the case of *O*-fucose carried by EGF12, where EV, MFNG, and RFNG resulted in a respective elongation of 4.5% (±2.3), 2.2% (±2.2), and 17.5% (±1.9) in CHO and 7.3% (±3.0), 14.0% (±3.4), and 23.1% (±3.4) in U2OS. When MFNG and RFNG were overexpressed together, the elongated form of *O*-fucose in EGF12 was 13.9% (±2.5) in CHO and 21.8% (±6.7) in U2OS, more similar to RFNG alone, and demonstrating dominance of RFNG over MFNG at EGF12 (Figure 6E and Figure 7E).

## 3. Discussion

Here, we showed that LFNG had a dominant effect over MFNG or RFNG on N1 activation by DLL1 in cell-based N1 signaling assays. Using mass spectral glycoproteomic analysis, we demonstrated that this dominance was due to the ability of LFNG to more extensively modify *O*-fucose residues present on EGF8 and 12, both of which play a key role in the activation of N1 by DLL1. Elimination of the *O*-fucose sites on EGF8 or 12 reduced the dominant effect of LFNG over MFNG and RFNG in most cases. In addition, we showed that both LFNG and MFNG had a dominant effect over RFNG on N1 activation by JAG1. Glycoproteomic analysis showed that both LFNG and MFNG modified *O*-fucose on EGF6, which inhibits JAG1-N1 activation, while RFNG alone did not. Co-expression of either LFNG or MFNG with RFNG resulted in modification of EGF6, reducing activation of N1 by JAG1. Elimination of the *O*-fucose site on EGF6 reduced the dominant effect of LFNG or MFNG over RFNG.

**Figure 8:**
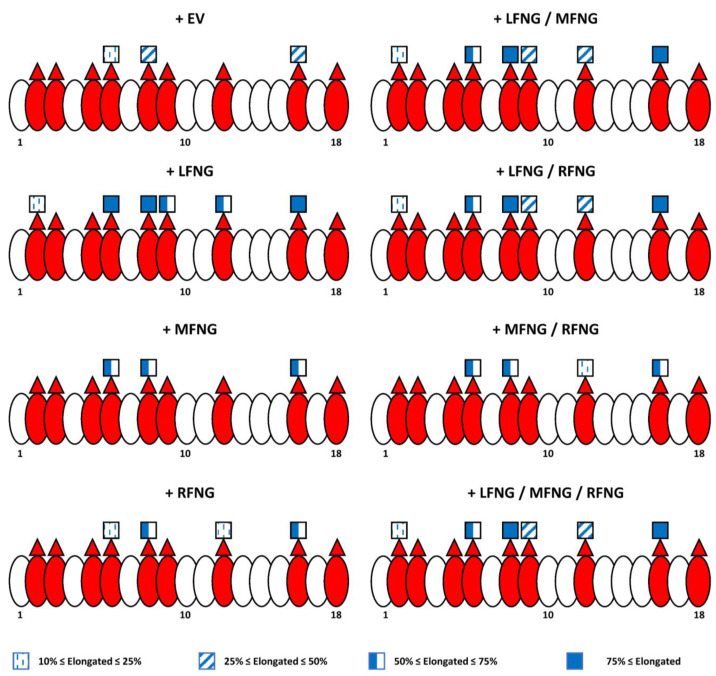
Summary diagram of the N1 EGF 1-18 *O*-fucosylation profile with different Fringe combinations. EGFs of the extracellular domain of the N1 receptor are represented by ovals. Red ovals represent EGFs containing the POFUT1 consensus sequence: C^2^XXXX(**S**/**T**)C^3^. Red triangles represent the presence of an *O*-linked fucose, and blue squares represent GlcNAc. Further elongation is not shown for simplicity. The percentage of GlcNAc modification of *O*-fucose is indicated by the shading of the GlcNAc (key below) using the average of the results obtained in U2OS and CHO cells. An elongation of less than 10% was considered negligible.

**Figure 9:**
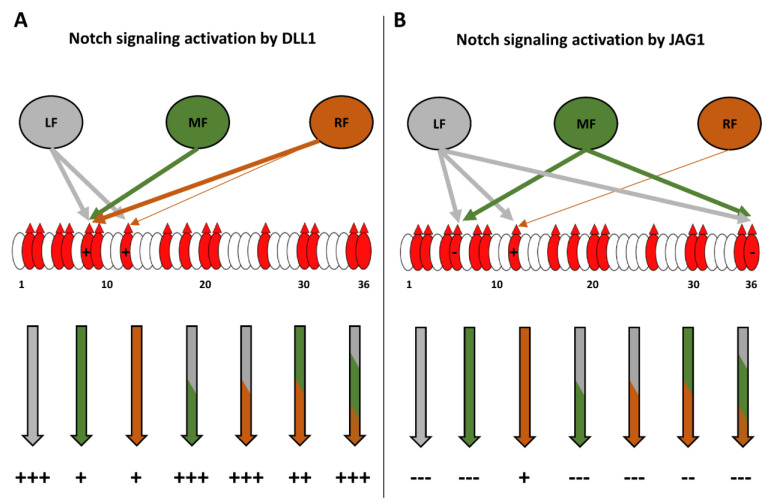
Summary diagram of the effect of the different Fringe combinations on the activation of the NOTCH1 pathway by DLL1 and JAG1. EGFs of the extracellular domain of the N1 receptor are represented by ovals, and red ovals represent EGFs containing the POFUT1 consensus sequence: C^2^XXXX(**S**/**T**)C^3^. Red triangles represent the presence of *O*-linked fucose. (**A**) LFNG (in grey) strongly modifies EGF8 and 12. This results in a strong activation of the N1 pathway by DLL1, whether LFNG is present alone or with MFNG and/or RFNG. MFNG (in green) modifies EGF8 but not EGF12, and RFNG (in orange) modifies EGF8 and slightly EGF12. Consequently, the presence of MFNG or RFNG alone or together results in a lower activation than LFNG of the N1 signaling induced by DLL1. (**B**) LFNG and MFNG strongly modify EGF6 and 36, inducing a strong inhibition of N1 signaling from JAG1 either alone, together or with RFNG. The ability of LFNG to modify EGF12 is not sufficient to prevent the inhibition induced by the elongation of *O*-fucoses in EGF6 and 36. RFNG slightly modifies EGF12, which slightly favors the activation of N1 receptor by JAG1 [4]. MFNG modifies EGF6 and potentially 36 with less efficiency than LFNG. Thus, when MFNG and RFNG are present together, the inhibition induced by MFNG is less intense than when it is alone or with LFNG.

Figure 8 summarizes our site-mapping data, and Figure 9 summarize the effects of single or multiple Fringes on N1 activity. In the context of N1 pathway activation by DLL1, the ability of LFNG to strongly modify EGF8 and 12 makes its effect dominant over that of MFNG, which only modifies EGF8, and over RFNG, which modifies EGF8 and weakly EGF12 (Figure 8 and Figure 9A). The effects of MFNG and RFNG are similar, despite the ability of RFNG to facilitate the activation of the N1 receptor by DLL1 by targeting the *O*-fucose carried by EGF12 more efficiently than MFNG (Figure 8 and Figure 9A). In the case of N1 pathway induction by JAG1, the ability of LFNG and MFNG to target the *O*-fucose of EGF6 and 36 makes their effect dominant over RFNG (Figure 8 and Figure 9B). However, the lower efficiency of MFNG modifying EGF6 and potentially EGF36 makes its dominance over RFNG less significant than that of LFNG. Moreover, the ability of RFNG to target EGF12 could slightly counteract JAG1-N1 inhibition by MFNG (Figure 8 and Figure 9B). Interestingly, the combination of multiple Fringes leads to a level of elongation similar to the elongation observed for the most efficient Fringe (usually LFNG) (Figure 8). Thus, our data suggest that there is no competition or interaction between LFNG, MFNG, and RFNG.

Several studies have revealed the expression of more than one Fringe within the same cell type [26,29,30,31,32,33]. Our data help to explain the results in some of these studies. For instance, a prior study showed that LFNG, MFNG, and RFNG are expressed in T cell progenitors and that the expression of all three Fringes is needed for optimal N1-dependent T cell development. Nonetheless, the expression of LFNG alone (double knockout of *Mfng* and *Rfng*) resulted in similar levels of thymocytes to that in WT mice, while the presence of MFNG or RFNG alone resulted in thymocyte levels similar to triple-Fringe KO mice [29]. *Lfng*-null mice, expressing only MFNG and RFNG, had slightly lower levels of thymocytes than the *Lfng*-only mice, but message levels for *Mfng* and *Rfng* were much higher than for *Lfng*. These results suggest that while all Fringes are needed for optimal T cell development, LFNG provides a higher level of N1 activation. Since DLL4 is the ligand activating N1 during T cell development [29], *O*-fucose elongation of EGF8 and 12 would likely be important for activation of N1. Of the three Fringes, LFNG most efficiently modifies these two sites, leading to higher activation of the N1 than that observed in the presence of MFNG or RFNG. Another study conducted on retinal endothelial cells showed that all three Fringes are expressed, although higher levels of mRNA for *Mfng* and *Rfng* than *Lfng* [31]. Loss of *Lfng* promotes angiogenic sprouting, corresponding to a reduction in Notch activation, despite the presence of MFNG and RFNG [31]. They suggest that LFNG action promotes Notch signaling by DLL4 to maintain the stalk phenotype of adjacent cells [31]. Thus, the fact that the loss of LFNG is sufficient to induce a decrease in Notch activation to promote angiogenesis highlights a dominance of the effect of LFNG over MFNG and RFNG, again likely by modifying EGF8 and 12 with more efficiency than the other two Fringes. A third study comparing naive CD4^+^ T cells between control and asthmatic rats revealed a strong increase in RFNG expression associated with a decrease in LFNG and MFNG. These variations are associated with an overactivation of the Notch pathway [30]. This suggests a decrease in the *O*-fucose elongation of EGF6 and potentially 36 targeted by LFNG and MFNG, which would weaken inhibition of the Notch pathway induced by Jagged ligands. Moreover, the increase of RFNG would increase the elongation of the *O*-fucose of EGF12, further promoting the activation of the Notch pathway by either Delta or Jagged ligands. These two cumulative phenomena would lead to overactivation of the Notch pathway. Overexpression of LFNG in naive CD4^+^ T cells from asthmatic rats partially rescued the activation of the Notch pathway [30], suggesting that LFNG is dominant over RFNG in this context. Interestingly, overexpression of MFNG did not have the same effect, suggesting other factors are involved in Notch regulation in this system.

Our analysis of the effects of individual Fringes on N1 activation by DLL1 or JAG1 revealed that Fringes have similar effects in NIH3T3 and CHO cells but different effects in U2OS cells. In particular, MFNG expression enhanced DLL1-N1 activation in both NIH3T3 and CHO cells but not in U2OS cells, and inhibition of JAG1-N1 activation by LFNG was less efficient in U2OS cells. This raised the possibility that some Fringes were endogenously expressed at different levels in U2OS cells in comparison to NIH3T3 cells. Indeed, we detected a higher relative level of MFNG transcripts expressed in U2OS cells than NIH3T3, which may explain why expression of exogenous MFNG has no effect on DLL1-N1 activation in U2OS cells. This was confirmed by mass spectral analysis of N1 EGF1-18 in the absence of exogenous Fringes, which revealed that NIH3T3 and CHO cells had very little Fringe modification, while U2OS cells showed significant elongation of *O*-fucose on EGF6, 8, and 16 (Figure 3E). This pattern of modification was also observed when MFNG alone was expressed in CHO cells (Figure 3C). Thus, exogenous expression of MFNG enhanced modification of EGF6, 8, and 16 in U2OS cells, helping to explain why MFNG does not enhance DLL1-N1 activation in U2OS cells. The endogenous modification of *O*-fucose on EGF6 in U2OS cells also helps to explain why exogenous LFNG inhibits JAG1-N1 less efficiently in those cells. The lower level of inhibition induced by LFNG compared to MFNG expression can be explained by LFNG’s ability to modify the *O*-fucose of EGF12 (Figure 3B,F) contrary to MFNG in U2OS cells (Figure 7E), favoring the activation of N1 by JAG1 [4].

Surprisingly, our qRT-PCR analysis also showed that both NIH3T3 cells and U2OS cells expressed LFNG (and RFNG) mRNA at higher relative levels than MFNG mRNA. This was not reflected in the mass spectral analysis of N1 EGF1-18 in the absence of exogenous Fringes in these cells, especially with respect to modification of *O*-fucose on EGF2, 9, and EGF12 (Figure 8). These sites were poorly modified in U2OS cells in the absence of exogenous Fringes (Figure 3E) but were modified by exogenous LFNG in both U2OS and CHO cells (Figure 3B,F). Thus, the relative mRNA levels for MFNG and LFNG in U2OS cells did not accurately reflect the Fringe modification pattern seen in our mass spectral analysis. This suggests that factors other than mRNA levels (micro RNAs, protein degradation rates) may play important roles in the resulting glycan modifications in different cell types.

Our mass spectral analyses of N1 EGF1-18 showed that most of the *O*-fucose residues previously shown to be modified by LFNG, MFNG, or RFNG [4,8] were also modified in our study (Figure 3 and Figure 8). Surprisingly, here, we showed that the *O*-fucose on EGF16 can be modified by all three Fringes, which was not previously known (Figure 3 and Figure 8). The *O*-fucosylated threonine in the peptide from EGF16 analyzed in the earlier study (4) was very close to the *n*-terminus of the detected peptide (G**T**CQDRDNSYLC), and modification of the *O*-fucose may have interfered with protease digestion, making it difficult to detect elongation at that site. It will be interesting to determine the effect of elongation at EGF16 on the activation of the N1 receptor by its different ligands. In addition, we observed that MFNG did not modify the *O*-fucose on EGF9 (Figure 3 and Figure 8), contrary to what was previously shown [4]. It is not clear why we see this difference, but there are a number of possible reasons. The previous data were obtained using N1 EGF1-36 (rather than N1 EGF1-18) expressed in HEK293T cells (rather than CHO or U2OS cells), and the peptides were analyzed on a three-dimensional ion trap instrument as opposed to the Orbitrap used in this study. In addition, a different peptide was detected in both analyses. Our data also showed strong differences in the elongation of N1 *O*-fucose within the same cell line with or without Fringe overexpression (Figure 3).

The *O*-fucose residues on EGF2, 9, and 12 seem to be more difficult to elongate compared to EGF6, 8, and 16, suggesting a different affinity of Fringes for some N1 EGF repeats. The expression of chimeric N1 with an inversion of EGF12 and 16 or 8 would perhaps allow us to determine if the strong affinity of Fringes for *O*-fucose on one EGF repeat compared to another is due to different accessibility associated with their location or to a particularity within their respective amino acid sequences. Elongation of *O*-fucoses by Fringe is extremely specific and is not limited to the presence of an *O*-fucose. Studies have shown that Fringes only modify *O*-fucose on correctly folded EGFs in vitro [40], and that *O*-fucose residues linked to different domains, such as thrombospondin type 1 repeats (TSRs), are not modified by Fringes [41]. In addition, residues in a single EGF sequence affect modification by Fringes. A single EGF repeat from clotting factor 9 expressed in CHO cells bears the *O*-fucose tetrasaccharide, while a highly homologous EGF repeat from clotting factor 7 expressed in the same cells is modified by *O*-fucose monosaccharide [42]. Mutation of two amino acids of the factor 9 EGF repeat to the corresponding residues from factor 7 significantly reduces elongation of *O*-fucose. These results suggest that alteration of a few residues in a single EGF repeat can affect the ability of Fringe to modify *O*-fucose [42]. Finally, Fringes have different activities toward *O*-fucosylated EGF repeats in vitro. For instance, the specific activity of LFNG for modifying *O*-fucosylated N1 EGF26 is 6 times higher than RFNG and 150 times higher than MFNG [42]. Consequently, the observed dominance of LFNG over MFNG and RFNG could be correlated to the higher enzymatic activity of LFNG compared to MFNG and RFNG.

## 4. Materials and Methods

### 4.1. Plasmids

Plasmids encoding mouse NOTCH1 (N1) EGF1-18 with C-terminal Myc-His6 tags (pSecTag2-Hygro, Invitrogen) were described previously [6,40]. The plasmid encoding full-length mouse NOTCH1 (pcDNA1-N1-myc) was generously provided by Dr. Jefferey Nye [43]. Mutations in the *O*-fucose modification sites of EGF6, 8, 12, and 36 (T232V, T311V, T466V, and T1402V, respectively) were described previously [4]. Fringe-expressing plasmids APtag2 [EV], [LFNG], [MFNG], or [RFNG] were previously described [20]. The TP1-1 luciferase reporter construct (Ga981-6) was a gift from Dr. Georg Bornkamm (Munich, Germany), and the gWIZ β-galactosidase construct was from Gene Therapy Systems (San Diego, CA, USA).

### 4.2. Cell Culture

NIH3T3 (NIH3T3 CRL-1658), CHO Pro5, and U2OS were obtained from the American Type Culture Collection (Manassas, VA, USA) and L cells stably expressing Jagged1 (JAG1) or Delta-like 1 (DLL1) were a kind gift of Dr. Gerry Weinmaster (UCLA). NIH3T3 and U2OS cells were grown in Dulbecco’s modified Eagle’s medium (Hyclone) and CHO in Minimum Essential Medium α without nucleosides (Gibco), both supplemented with 10% bovine calf serum at 37 °C in a humidified incubator at 5% CO_2_.

### 4.3. RNA Isolation and qRT-PCR Analysis

NIH3T3 and U2OS cells were harvested, flash-frozen in liquid nitrogen, and stored at −80 °C until use. Total RNA isolation and cDNA synthesis on four biological replicates was carried out as described previously [44]. The qRT-PCR reactions were performed in triplicate for each gene analyzed using primer pairs listed in Tables S1 and S2. Amplification conditions and data analysis was performed as described previously [45]. Briefly, Ct values for each gene were normalized with the control gene, GAPDH, prior to calculation of relative transcript abundance.

### 4.4. Cell-Based Co-Culture N1 Activation Assay

NIH3T3, CHO, and U2OS cells (0.5 × 10^5^) were seeded in a 24-well tissue culture plate for 24 (NIH3T3 and U2OS) or 48 h (CHO) according to their growth rate. Complete media was removed, cells were washed once with 1× PBS, and DMEM (NIH3T3 and U2OS) or α-MEM (CHO) media without serum was added. The cells were then co-transfected with 0.2 μg of WT or mutant pcDNA1 [N1], 0.1 μg of APtag2 [EV], [LFNG], [MFNG], or [RFNG] plasmid (the total amount of APtag2 plasmid never exceeds 0.1 μg with 0.05 μg or 0.033 μg of each Fringe depending on the combinations used), 0.2 μg of TP1-1 luciferase reporter construct, and 0.1 μg of gWIZ β-galactosidase construct for transfection efficiency normalization using PEI (with a PEI/DNA ratio of 6/1) in 20μL OPTI-MEM. The Fringe/Notch1 plasmid ratio of 0.5 was previously determined to provide optimal effects on signaling [8]. After 4 h, media was removed, cells were washed once with 1X PBS, and DMEM (NIH3T3 and U2OS) or α-MEM (CHO) media with serum was added. L cells stably expressing JAG1 or DLL1 were added on the transfected cells at a density of 1.5 × 10^5^ (NIH3T3 and CHO) or 2 × 10^5^ (U2OS) cells/well for 24 h. Cells were lysed, and luciferase assays were performed based on the manufacturer’s instructions (Luciferase Assay System, Promega, Madison, WI, USA) as described previously [4,8,46].

### 4.5. Protein Expression and Purification

For each replicate and condition, eight 10 cm dishes of approximately 90% confluent NIH3T3, CHO, or U2OS cells were transiently transfected. Complete media was removed, cells were washed once with 1X PBS, and DMEM (NIH3T3 and U2OS) or α-MEM (CHO) media without serum was added. Cells were transfected with 5 μg pSecTag [N1 EGF1-18] plasmid and 2.5 μg of APtag2 [EV], [Lfng], [Mfng], or [Rfng] plasmid per 10 cm dish (with a PEI/DNA ratio of 4/1) in 500 μL OPTI-MEM. Media was collected after approximately 5 days, centrifuged at 4000× *g* for 15 min, and then syringe filtered with a 0.45 μm syringe filter. Then, 5 M NaCl and 1 M imidazole were added to a final concentration of 500 mM and 10 mM, respectively. For purification, a 150–200 μL Ni-NTA agarose bead volume (300–400 μL 50% slurry) was used (Qiagen). Wash buffer consisted of 1 M NaCl and 20 mM imidazole in 1× TBS. Proteins were eluted using 250 mM imidazole in 1× TBS.

### 4.6. Mass Spectral Analysis

First, 10 μL of purified protein was denatured and reduced using 10 μL of reducing buffer containing 8 M Urea, 400 mM ammonium bicarbonate, and 10 mM TCEP (trypsin and chymotrypsin digestion) or 8 M urea, 50 mM Tris-HCl, pH 8, and 10 mM TCEP (V8 digestion) at 50 °C for 5 min. Alkylation was performed at room temperature in the dark with 100 mM iodoacetamide in 50 mM Tris-HCl for 30 min. Then, 45 μL of mass spectral grade water (trypsin and chymotrypsin digestion) or 300 mM diammonium phosphate solution (V8 digestion) were added to each sample. Trypsin (30 ng), chymotrypsin (50 ng), or V8 (30 ng) was added, and samples were incubated in a 37 °C water bath for 20 (trypsin and V8) or 1 h (chymotrypsin). Next, 7 μL of 5% formic acid were added and samples were desalted with Millipore C18 Zip Tip Pipette Tips. After elution in 50% acetonitrile, 0.1% acetic acid, samples were diluted to 25% acetonitrile, and 0.1% formic acid. Approximately 10 ng of each sample were injected on a Q-Exactive Plus Orbitrap mass spectrometer (Thermo Fisher, Waltham, MA, USA) with an Easy nano-LC HPLC system with a C18 EasySpray PepMap RSLC C18 column (50 μm × 15 cm, Thermo Fisher Scientific, Waltham, MA, USA). A 30 min binary gradient solvent system (Solvent A: 5% acetonitrile, 0.1% formic acid in water and Solvent B: 80% acetonitrile, 0.1% formic acid in water) with a constant flow of 300 nL/min was used. Positive polarity mode was used with a *m/z* range of 400–2000 at a resolution of 70,000 and automatic gain control set to 1 × 10^6^. Higher energy collisional dissociation-tandem mass spectrometry (HCD-MS/MS) was used on the top 10 precursor ions in each full scan (collision energy set to 27%, 1 × 10^5^ gain control, isolation window *m/z* 1.2, dynamic exclusion enabled, and 17,500 fragment resolution). PMI-Byonic (v.2.10.5) and Proteome Discoverer (v2.1) were used to identify peptides. Fixed modifications: Carbamidomethyl +57.021464 at C. Variable modifications: Oxidation +15.994915 at M,H,N,D, Deamidated +0.984016. Glycoforms searched: unmodified peptide, modified peptide with *O*-fucose, modified peptide with *O*-fucose and HexNAc, modified peptide with *O*-fucose, HexNAc, and hexose or modified peptide with *O*-fucose, HexNAc, hexose, and NeuAc. All these glycoforms were searched for in association with the presence or absence of *O*-hexose, *O*-hexose and pentose, or *O*-hexose, pentose, and pentose (Table S3). Precursor and fragment mass tolerance was set to 20 ppm. Four missed cleavages were allowed. Protein and peptide false discovery rates were set to a threshold of 1% and calculated in Byonic software version 2.10.5 (Protein Metrics) using the 2-dimensional target decoy strategy as described [47]. The extracellular part of the murine N1 receptor containing EGF1 to 18 (Q01705, 18 April 2012—v3) was used as a database. Xcalibur Qual Browser (v2.0.3) was used to generate EICs for all identified *O*-fucosylated peptides. For each peptide, the area under the curve was calculated for each peak corresponding to searched glycoforms. Relative abundance was calculated by comparing the area under the curve for a single glycoform to the total area under curve for all searched glycoforms of a specific peptide. MS/MS spectra for each glycopeptide analyzed are shown in Figure S7.

### 4.7. Statistical Analysis

All experiments were performed in biological triplicates or more and results were reported as the means ± standard deviation (SD). Statistical significance was determined using one-way ANOVA. Significance levels: (***) for *p* < 0.005, (**) for *p* < 0.001, (*) for *p* < 0.05.

## Data Availability

The mass spectrometry proteomics data have been deposited to the ProteomeXchange Consortium via the PRIDE [48] partner repository (https://www.ebi.ac.uk/pride/ accessed on 28 July 2021) with the data set identifier PXD027619. Table S54 provides a description of the files in the PRIDE repository.

## Supplementary Materials

The following are available online at https://www.mdpi.com/article/10.3390/molecules26195942/s1, Table S1: List of the mouse primers used for qRT-PCR analyses (NIH3T3); Table S2: List of the human primers used for qRT-PCR analyses (U2OS); Tables S3–S54; Figure S1: O-fucose glycoforms of N1 receptor in the absence of exogenous Fringe; Figure S2: O-fucose glycoforms of N1 receptor in presence of Fringe in CHO and U2OS cell lines; Figure S3: Elongation of N1 EGF18 in CHO and U2OS cell lines; Figure S4: Effect of the mutation of EGF8 or 12 O-fucosylation sites on Fringe dominance associated with N1 signaling induced by DLL1; Figure S5: Effect of the mutation of EGF6 or 36 O-fucosylation sites on Fringe dominance associated with N1 signaling induced by JAG1; Figure S6: O-fucosylation profile of EGF3 and 5 of the N1 receptor in the presence of LFNG, MFNG and RFNG; Figure S7: MS/MS spectra for O-fucosylated peptides from EGF1-18.
