# Supplementary material for: Modulation of the NOTCH1 Pathway by LUNATIC FRINGE Is Dominant over That of MANIC or RADICAL FRINGE
Source: Molecules. 2021 Sep 30;26(19):5942. doi: 10.3390/molecules26195942 (PMC8512825; doi:10.3390/molecules26195942)
Supplement: Supplementary file 1 [file molecules-26-05942-s001.zip › molecules-1348141-supplementary.pdf]

SUPPLEMENTARY MATERIALS

Modulation of the NOTCH1 pathway by LUNATIC FRINGE is dominant over that of MANIC or RADICAL FRINGE

Florian Pennarubia, Alison V. Nairn, Megumi Takeuchi, Kelley W. Moremen, Robert S Haltiwanger[*]

Complex Carbohydrate Research Center, Department of Biochemistry and Molecular Biology, University of Georgia, Athens, GA 30602, USA.

[*]Corresponding author: Robert S. Haltiwanger

Contents:
Tables S1-S54
Figures S1-S7

Gene Name	Gene ID	Accession #	Forward Primer (5' to 3')	Reverse Primer (5' to 3')
Pofut1	140484	NM_080463	CTCTCACCATGACCATGTGC	CTGGCATTCAGTGCTCTCAC
Lfng	16848	NM_008494	TTCATCGCCGTCAAGACCACC	CTCCTTGTGGCGCGAGATCC
Mfng	17305	NM_008595	GCCCTCTTTACCCTCCTGTGC	CGCGCCCTGTATCATCCTCTG
Rfng	19719	NM_009053	GCCTGATGACTGCACTGTGGG	CAGGTGCGAGTGGAACAGGG
B4galt2	53418	NM_017377	GCCGCTATTCTCCACCCGAC	GGTGCTCCCGGTGTCTAAAGG
St3gal4	20443	NM_009178	GCTCCTGTGGCTGGCTACG	GGGTCAAAGTGGGCCGACTC
St6gal1	20440	NM_145933	TGCGTGTGGAAGAAAGGGAGC	CTCCTGGCTCTTCGGCATCTG
St6gal2	240119	NM_172829	AGCAATCCTGCGGCACCTATG	CCGCTGCTTGCCCTGTAGAG

Table S1: List of the mouse primers used for qRT-PCR analyses (NIH3T3).

Gene Name	Gene ID	Accession #	Forward Primer (5' to 3')	Reverse Primer (5' to 3')
POFUT1	23509	NM_015352	CCCCCTCAGGATGACTATGT	GACCTCACCCAGAGCTTCAC
LFNG	3955	NM_001040167	CGTCTACGTCGGCAAGC	CACCTTGTTCTCGCTGACC
MFNG	4242	NM_002405	GGCTGGTACAGTTCTGGTTTG	TCAAAGCCAGTTTGCGATT
RFNG	5986	NM_002917	ACAGTTGGCTACATCGTGGA	CTCCAGGTGAGAGTGGAAGAG
B4GALT2	8704	NM_003780	CAAGGCTGTGCTCCTTCTCT	CTGGGCGTAGACGTCAAAGT
ST3GAL4	6484	NM_006278	CGTCCTGGTAGCTTTCAAGG	GCACCCGCTTCTTATCACTC
ST6GAL1	6480	NM_003032	TATCTGCCCAAGGAGAGCAT	GACGACACAACAGCACACCT
ST6GAL2	84620	NM_032528	CTGTACCGGCTCTGGAAGG	ATCCTTCATCGCCTTCTGC

Table S2: List of the human primers used for qRT-PCR analyses (U2OS).

Table S3: List of searched peptides and glycoforms used for EICs generation and quantification

Table S4: Trypsin digestion of mN1 EGF1-18 + EV expressed in CHO analyzed for Figure 3, 6, S1, S3 and S6.

Table S5: V8 digestion of mN1 EGF1-18 + EV expressed in CHO analyzed for Figure 3, 6, S1, S3 and S6.

Table S6: Chymotrypsin digestion of mN1 EGF1-18 + EV expressed in CHO analyzed for Figure 3, 6, S1, S3 and S6.

Table S7: Trypsin digestion of mN1 EGF1-18 + LFNG expressed in CHO analyzed for Figure 3, 6, S2 ,S3 and S6.

Table S8: V8 digestion of mN1 EGF1-18 + LFNG expressed in CHO analyzed for Figure 3, 6, S2 ,S3 and S6.

Table S9: Chymotrypsin digestion of mN1 EGF1-18 + LFNG expressed in CHO analyzed for Figure 3, 6, S2 ,S3 and S6.

Table S10: Trypsin digestion of mN1 EGF1-18 + MFNG expressed in CHO analyzed for Figure 3, 6, S2 ,S3 and S6.

Table S11: V8 digestion of mN1 EGF1-18 + MFNG expressed in CHO analyzed for Figure 3, 6, S2 ,S3 and S6.

Table S12: Chymotrypsin digestion of mN1 EGF1-18 + MFNG expressed in CHO analyzed for Figure 3, 6, S2 ,S3 and S6.

Table S13: Trypsin digestion of mN1 EGF1-18 + RFNG expressed in CHO analyzed for Figure 3, 6, S2 ,S3 and S6.

Table S14: V8 digestion of mN1 EGF1-18 + RFNG expressed in CHO analyzed for Figure 3, 6, S2 ,S3 and S6.

Table S15: Chymotrypsin digestion of mN1 EGF1-18 + RFNG expressed in CHO analyzed for Figure 3, 6, S2 ,S3 and S6.

Table S16: Trypsin digestion of mN1 EGF1-18 + LFNG + MFNG expressed in CHO analyzed for Figure 6, S3 and S6.

Table S17: V8 digestion of mN1 EGF1-18 + LFNG + MFNG expressed in CHO analyzed for Figure 6, S3 and S6.

Table S18: Chymotrypsin digestion of mN1 EGF1-18 + LFNG + MFNG expressed in CHO analyzed for Figure 6, S3 and S6.

Table S19: Trypsin digestion of mN1 EGF1-18 + LFNG + RFNG expressed in CHO analyzed for Figure 6, S3 and S6.

Table S20: V8 digestion of mN1 EGF1-18 + LFNG + RFNG expressed in CHO analyzed for Figure 6, S3 and S6.

Table S21: Chymotrypsin digestion of mN1 EGF1-18 + LFNG + RFNG expressed in CHO analyzed for Figure 6, S3 and S6.

Table S22: Trypsin digestion of mN1 EGF1-18 + MFNG + RFNG expressed in CHO analyzed for Figure 6, S3 and S6.

Table S23: V8 digestion of mN1 EGF1-18 + MFNG + RFNG expressed in CHO analyzed for Figure 6, S3 and S6.

Table S24: Chymotrypsin digestion of mN1 EGF1-18 + MFNG + RFNG expressed in CHO analyzed for Figure 6, S3 and S6.

Table S25: Trypsin digestion of mN1 EGF1-18 + LFNG + MFNG + RFNG expressed in CHO analyzed for Figure 6, S3 and S6.

Table S26: V8 digestion of mN1 EGF1-18 + LFNG + MFNG + RFNG expressed in CHO analyzed for Figure 6, S3 and S6.

Table S27: Chymotrypsin digestion of mN1 EGF1-18 + LFNG + MFNG + RFNG expressed in CHO analyzed for Figure 6, S3 and S6.

Table S28: Trypsin digestion of mN1 EGF1-18 + EV expressed in U2OS analyzed for Figure 3, 7, S1, S3 and S6.

Table S29: V8 digestion of mN1 EGF1-18 + EV expressed in U2OS analyzed for Figure 3, 7, S1, S3 and S6.

Table S30: Chymotrypsin digestion of mN1 EGF1-18 + EV expressed in U2OS analyzed for Figure 3, 7, S1, S3 and S6.

Table S31: Trypsin digestion of mN1 EGF1-18 + LFNG expressed in U2OS analyzed for Figure 3, 7, S2 ,S3 and S6.

Table S32: V8 digestion of mN1 EGF1-18 + LFNG expressed in U2OS analyzed for Figure 3, 7, S2 ,S3 and S6.

Table S33: Chymotrypsin digestion of mN1 EGF1-18 + LFNG expressed in U2OS analyzed for Figure 3, 7, S2 ,S3 and S6.

Table S34: Trypsin digestion of mN1 EGF1-18 + MFNG expressed in U2OS analyzed for Figure 3, 7, S2 ,S3 and S6.

Table S35: V8 digestion of mN1 EGF1-18 + MFNG expressed in U2OS analyzed for Figure 3, 7, S2 ,S3 and S6.

Table S36: Chymotrypsin digestion of mN1 EGF1-18 + MFNG expressed in U2OS analyzed for Figure 3, 7, S2 ,S3 and S6.

Table S37: Trypsin digestion of mN1 EGF1-18 + RFNG expressed in U2OS analyzed for Figure 3, 7, S2 ,S3 and S6.

Table S38: V8 digestion of mN1 EGF1-18 + RFNG expressed in U2OS analyzed for Figure 3, 7, S2 ,S3 and S6.

Table S39: Chymotrypsin digestion of mN1 EGF1-18 + RFNG expressed in U2OS analyzed for Figure 3, 7, S2 ,S3 and S6.

Table S40: Trypsin digestion of mN1 EGF1-18 + LFNG + MFNG expressed in U2OS analyzed for Figure 7, S3 and S6.

Table S41: V8 digestion of mN1 EGF1-18 + LFNG + MFNG expressed in U2OS analyzed for Figure 7, S3 and S6.

Table S42: Chymotrypsin digestion of mN1 EGF1-18 + LFNG + MFNG expressed in U2OS analyzed for Figure 7, S3 and S6.

Table S43: Trypsin digestion of mN1 EGF1-18 + LFNG + RFNG expressed in U2OS analyzed for Figure 7, S3 and S6.

Table S44: V8 digestion of mN1 EGF1-18 + LFNG + RFNG expressed in U2OS analyzed for Figure 7, S3 and S6.

Table S45: Chymotrypsin digestion of mN1 EGF1-18 + LFNG + RFNG expressed in U2OS analyzed for Figure 7, S3 and S6.

Table S46: Trypsin digestion of mN1 EGF1-18 + MFNG + RFNG expressed in U2OS analyzed for Figure 7, S3 and S6.

Table S47: V8 digestion of mN1 EGF1-18 + MFNG + RFNG expressed in U2OS analyzed for Figure 7, S3 and S6.

Table S48: Chymotrypsin digestion of mN1 EGF1-18 + MFNG + RFNG expressed in U2OS analyzed for Figure 7, S3 and S6.

Table S49: Trypsin digestion of mN1 EGF1-18 + LFNG + MFNG + RFNG expressed in U2OS analyzed for Figure 7, S3 and S6.

Table S50: V8 digestion of mN1 EGF1-18 + LFNG + MFNG + RFNG expressed in U2OS analyzed for 7, S3 and S6.

Table S51: Chymotrypsin digestion of mN1 EGF1-18 + LFNG + MFNG + RFNG expressed in U2OS analyzed for Figure 7, S3 and S6.

Table S52: V8 digestion of mN1 EGF1-18 + EV expressed in NIH3T3 analyzed for Figure S1.

Table S53: Chymotrypsin digestion of mN1 EGF1-18 + EV expressed in NIH3T3 analyzed for Figure S1.

Table S54: Description of the files uploaded to the PRIDE repository.

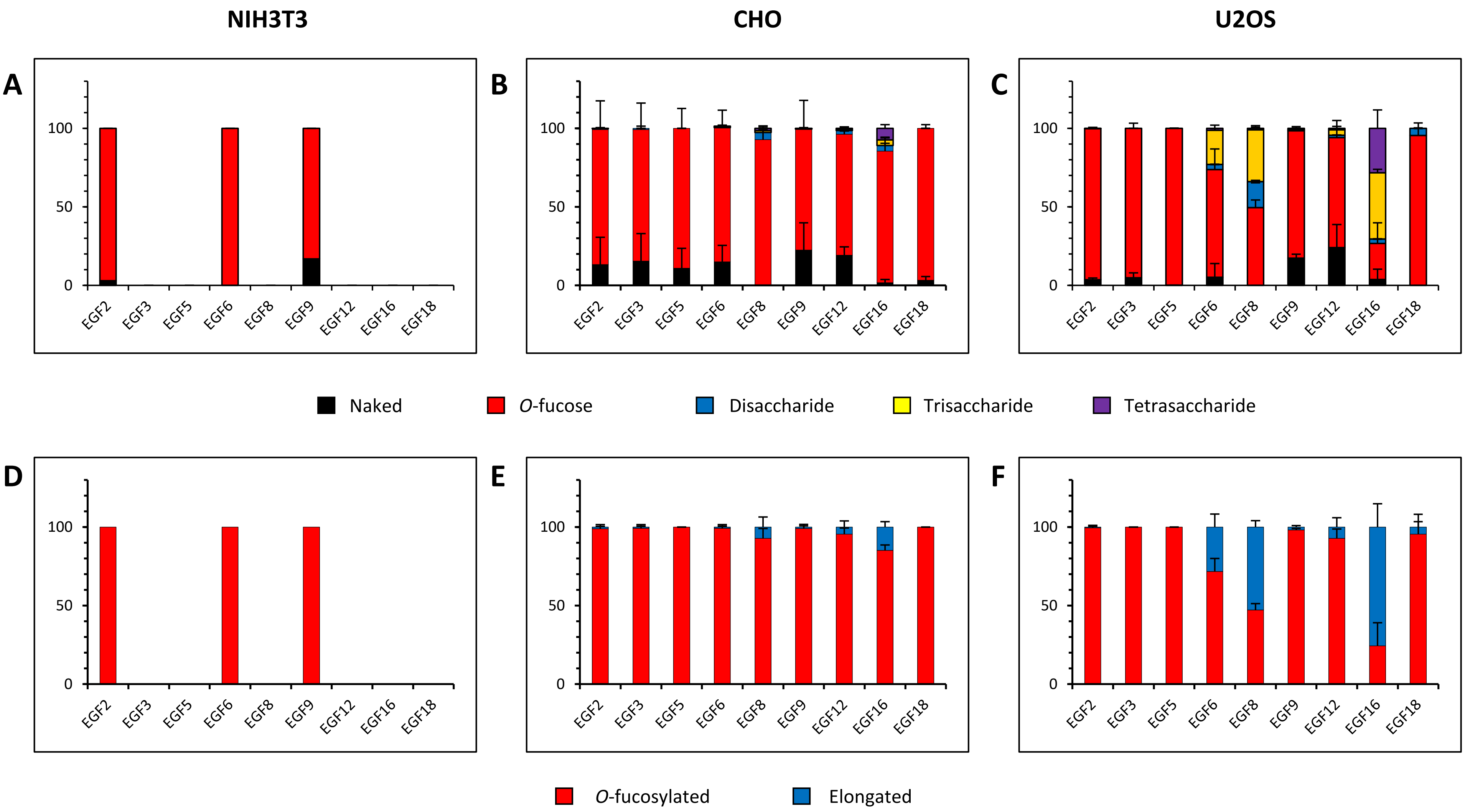

Figure S1: *O*-fucose glycoforms of N1 receptor in the absence of exogenous Fringe. (A-C) Quantification of the percentage of naked peptide (black), peptides modified by a monosaccharide (red), disaccharide (blue), trisaccharide (yellow), tetrasaccharide (purple) *O*-fucose (D-F) Quantification of the percentage of peptides modified by a monosaccharide *O*-fucose (red) and elongated *O*-fucose (blue) by at least a GlcNAc. The Extracted Ion Chromatogram (EIC) of the Mass Spectrometry (MS) data of peptides derived from mouse N1 EGF1-18 overexpressed in (A, C) NIH3T3, (B, D) CHO and (C-F) U2OS were used for the quantification. Bar graph shows mean +/− SD ; three or four independent experiments (depending on the cell line) n = 3 to 4 were analyzed. The data used to generate the EICs are available in the Table S4 to S6 (CHO), S28 to 30 (U2OS), S52 and S53 (NIH3T3). The ions used to generate the EICs are in Table S3.

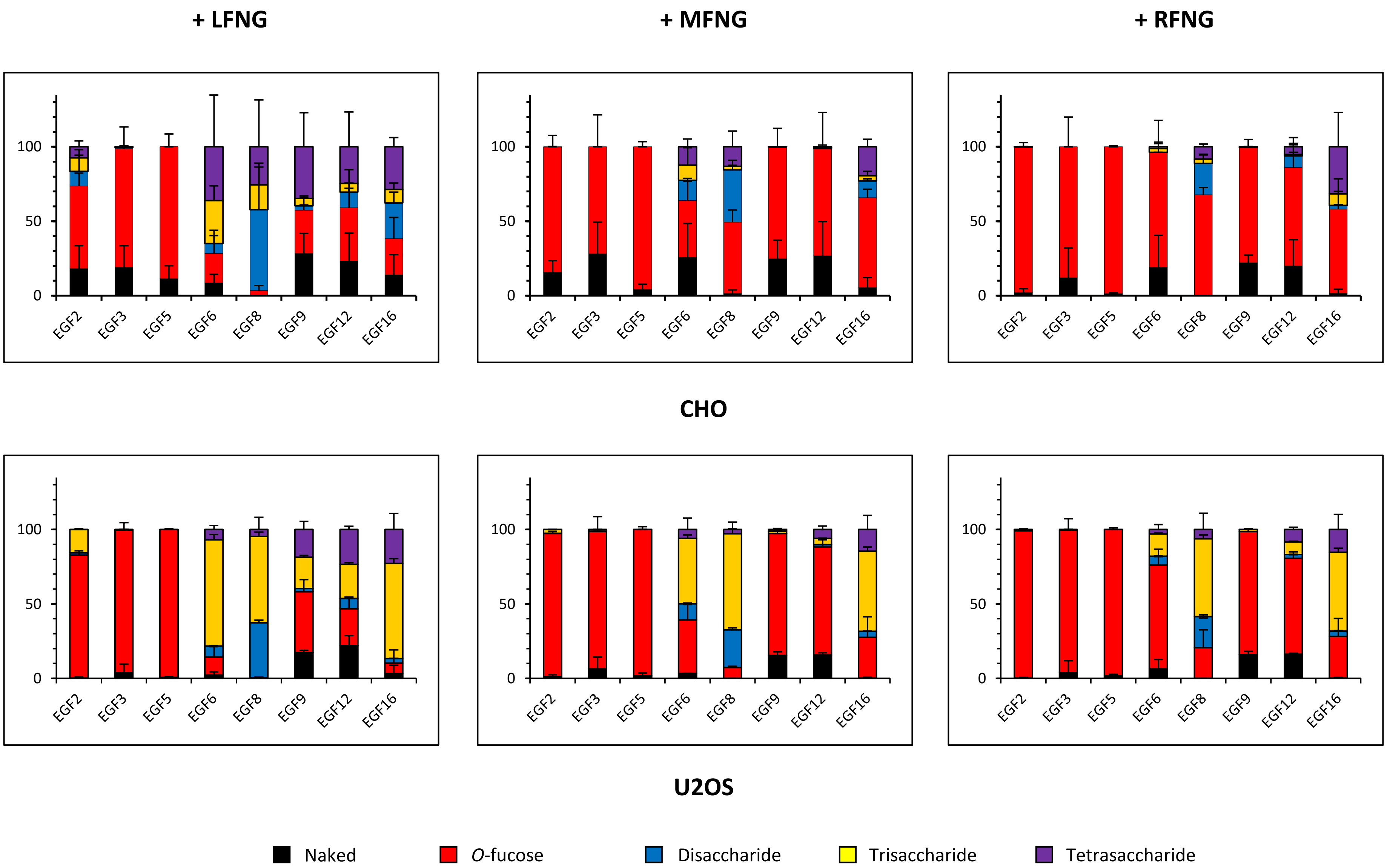

Figure S2: *O*-fucose glycoforms of N1 receptor in presence of Fringe in CHO and U2OS cell lines. Quantification of the percentage of naked peptide (black), peptides modified by a monosaccharide (red), disaccharide (blue), trisaccharide (yellow), tetrasaccharide (purple) *O*-fucose. The Extracted Ion Chromatogram (EIC) of the Mass Spectrometry (MS) data of peptides derived from mouse N1 EGF1-18 overexpressed in CHO (top) and U2OS (bottom) were used for the quantification. Bar graph shows mean +/− SD ; three or four independent experiments (depending on the cell line) n = 3 to 4 were analyzed. The data used to generate the EICs are available in the Table S7 to S15 (CHO), S31 to S39 (U2OS). The ions used to generate the EICs are in Table S3.

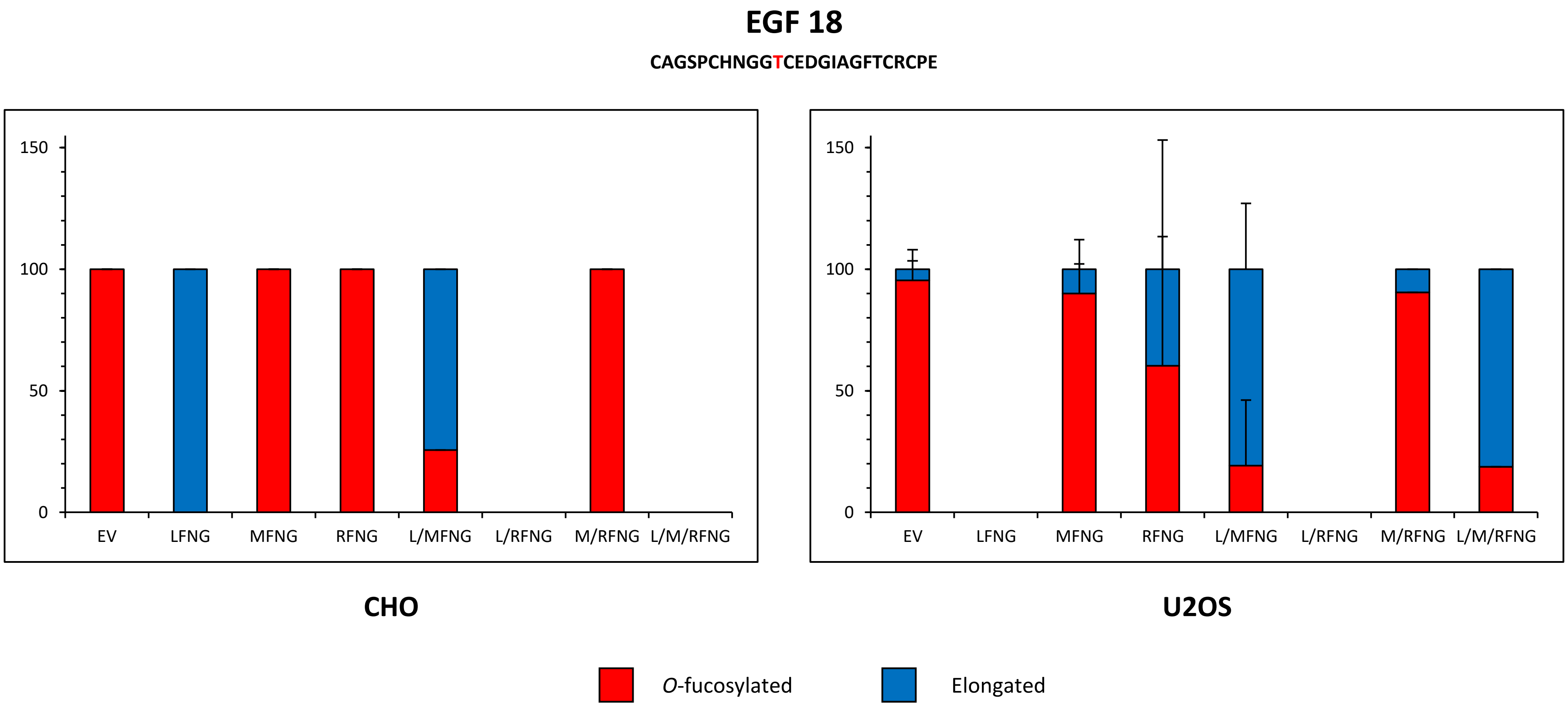

Figure S3: Elongation of N1 EGF18 in CHO and U2OS cell lines. Quantification of the percentage of peptides modified by a monosaccharide *O*-fucose (red) and elongated *O*-fucose (blue) by at least a GlcNAc. The Extracted Ion Chromatogram (EIC) of the Mass Spectrometry (MS) data of peptides derived from mouse N1 EGF1-18 overexpressed in CHO (left) and U2OS (right) were used for the quantification. Bar graph shows mean +/− SD ; one to three independent experiments (depending on the cell line and the condition) n = 1 to 3 were analyzed. The data used to generate the EICs are available in the Table S4 to S27 (CHO) and Table S28 to S51 (U2OS). The ions used to generate the EICs are in Table S3.

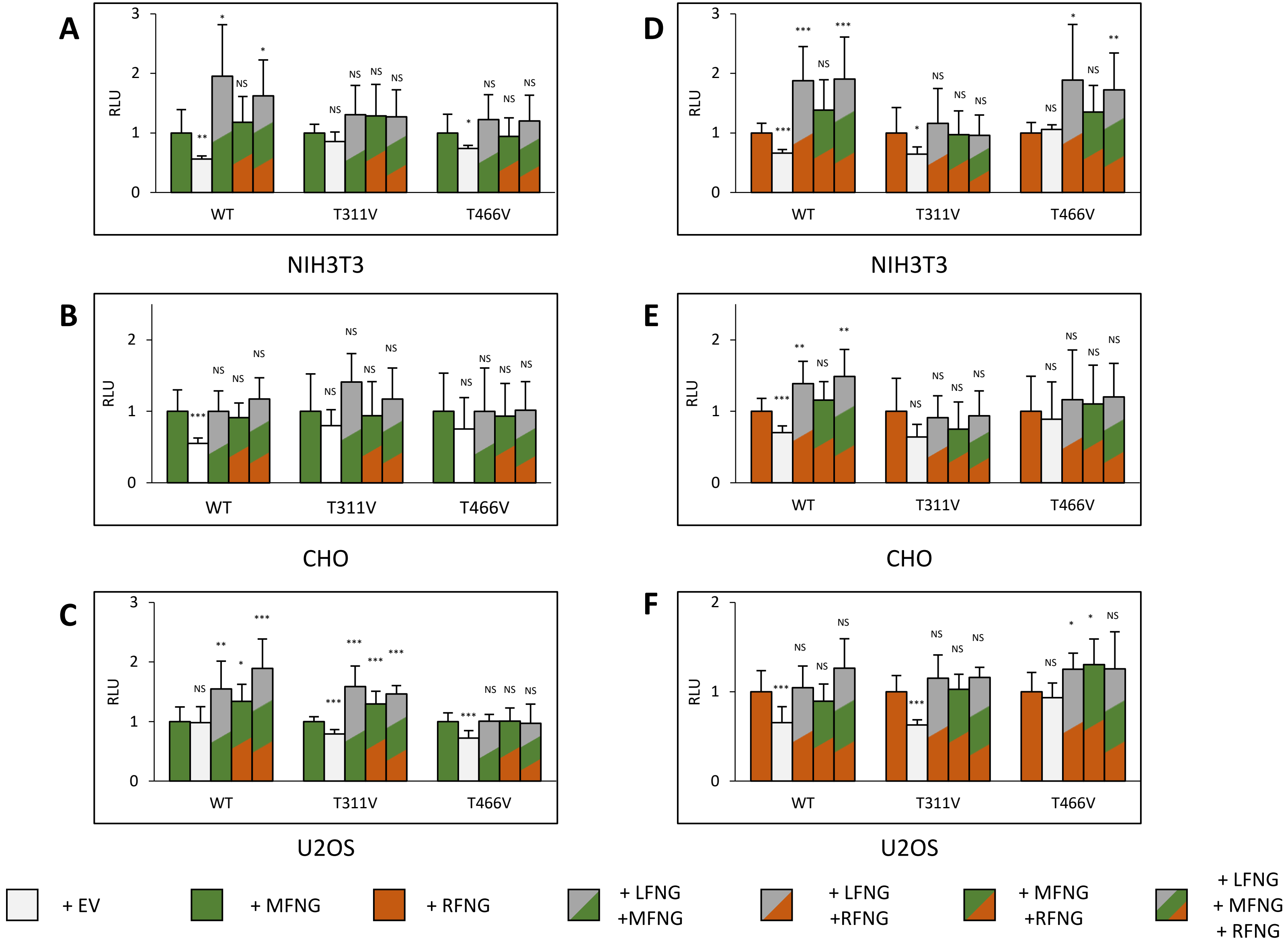

Figure S4: Effect of the mutation of EGF8 or 12 *O*-fucosylation sites on Fringe dominance associated with N1 signaling induced by DLL1. (A), (D) NIH3T3, **(B), (E)** CHO and **(C), (F)** U2OS were co-transfected with plasmids encoding full-length mouse wild-type (WT) N1 or mutated on EGF8 ($T^{311}V$) or 12 ($T^{466}V$) *O*-fucosylation site with empty vector (EV) or Lunatic Fringe (LFNG) and/or Manic Fringe (MFNG) and/or Radical Fringe (RFNG), then cell-based co-culture N1 activation assays was performed with L-cell stably overexpressed DLL1. Relative Luciferase units (RLU) compared **(A-C)** to MFNG or **(D-F)** to RFNG (normalized to 1 for each condition and cell line) were determined. Statistical significance was calculated using one-way ANOVA. Bar graph shows mean +/− SD; four or five independent experiments (depending on the cell line) n = 8 to 10 were analyzed (***, $p < 0.005$; **, $p < 0.01$; *, $p < 0.05$).

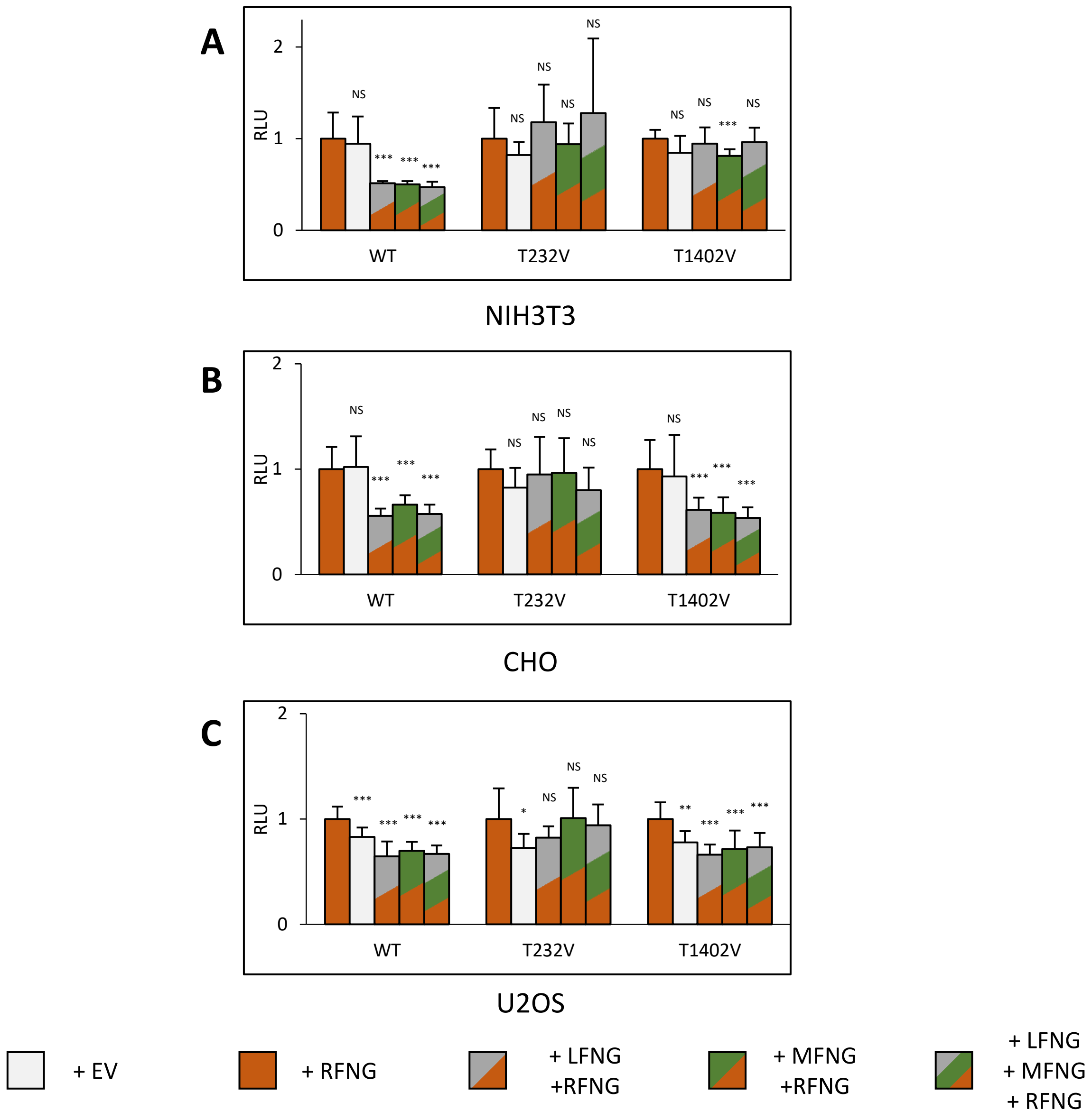

Figure S5: Effect of the mutation of EGF6 or 36 *O*-fucosylation sites on Fringe dominance associated with N1 signaling induced by JAG1. (A) NIH3T3, **(B)** CHO and **(C)** U2OS were co-transfected with plasmids encoding full-length mouse wild-type (WT) N1 or mutated on EGF6 ($T^{232}V$) or 36 ($T^{1402}V$) *O*-fucosylation site with empty vector (EV) or Lunatic Fringe (LFNG) and/or Manic Fringe (MFNG) and/or Radical Fringe (RFNG), then cell-based co-culture N1 activation assays was performed with L-cell stably overexpressed JAG1. Relative Luciferase units (RLU) compared to RFNG (normalized to 1 for each condition and cell line) were determined. Statistical significance was calculated using one-way ANOVA. Bar graph shows mean +/− SD; four or five independent experiments (depending on the cell line) n = 8 to 10 were analyzed (***, $p < 0.005$; **, $p < 0.01$; *, $p < 0.05$).

EGF 3

NGGTCDLLTLTEYK

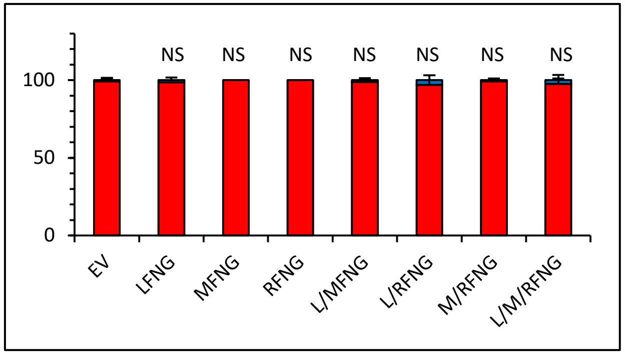

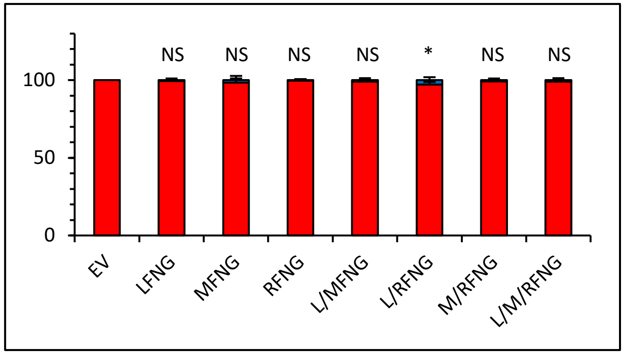

EGF3	LFNG	MFNG	RFNG	L/MFNG	L/RFNG	M/RFNG	L/M/RFNG
LFNG		NS	NS	NS	NS		NS
MFNG			NS	NS		NS	NS
RFNG					NS	NS	NS

EGF3	LFNG	MFNG	RFNG	L/MFNG	L/RFNG	M/RFNG	L/M/RFNG
LFNG		NS	NS	NS	NS		NS
MFNG			NS	NS		NS	NS
RFNG					*	NS	NS

EGF 5

ICRCPPGFHGPTCRQDVNECSQNPGLCRHGGTCHNEIGSY

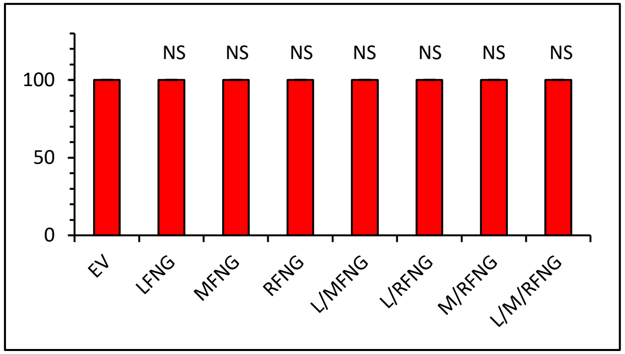

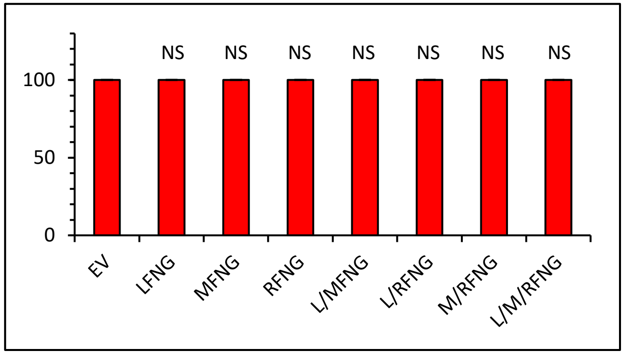

EGF5	LFNG	MFNG	RFNG	L/MFNG	L/RFNG	M/RFNG	L/M/RFNG
LFNG		NS	NS	NS	NS		NS
MFNG			NS	NS		NS	NS
RFNG					NS	NS	NS

EGF5	LFNG	MFNG	RFNG	L/MFNG	L/RFNG	M/RFNG	L/M/RFNG
LFNG		NS	NS	NS	NS		NS
MFNG			NS	NS		NS	NS
RFNG					NS	NS	NS

CHO **U2OS**

O-fucosylated Elongated

Figure S6: *O*-fucosylation profile of EGF3 and 5 of the N1 receptor in the presence of LFNG, MFNG and RFNG. Quantification of the percentage of peptides modified by a *O*-fucose (red) and elongated *O*-fucose (blue) by at least a GlcNAc. The Extracted Ion Chromatogram (EIC) of the Mass Spectrometry (MS) data of peptides derived from mouse N1 EGF1-18 overexpressed with EV, LFNG, MFNG, RFNG, LFNG + MFNG, LFNG + RFNG, MFNG + RFNG or LFNG + MFNG + RFNG in CHO and U2OS were used for the quantification. Statistical significance was calculated using one-way ANOVA. Bar graph shows mean +/− SD; three or four independent experiments (depending on the cell line) n = 3 to 4 were analyzed (***, $p < 0.005$; **, $p < 0.01$; *, $p < 0.05$). The data used to generate the EICs are available in the Table S4 to S27 (CHO) and Table S28 to S51 (U2OS). The ions used to generate the EICs are in Table S3.

Figure S7A

EGF 2 : ^{53}VGQRCQDSNPCLSTPCKNAGTCHVVDHGGTVDY^{85}

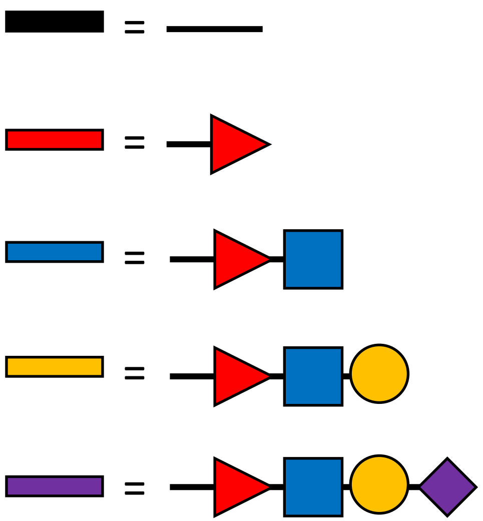

#1	b⁺	b²⁺	b³⁺	b⁴⁺	Seq.	y⁺	y²⁺	y³⁺	y⁴⁺	#2
1	100.07569	50.54148	34.03008	25.77438	V					33
2	157.09715	79.05222	53.03724	40.02975	G	4162.72164	2081.86446	1388.24540	1041.43587	32
3	285.15573	143.08150	95.72343	72.04439	Q	4105.70018	2053.35373	1369.23824	1027.18050	31
4	441.25684	221.13206	147.75713	111.06967	R	3977.64160	1989.32444	1326.55205	995.16586	30
5	601.28749	301.14738	201.10068	151.07733	C-Carbami...	3821.54049	1911.27388	1274.51835	956.14058	29
6	729.34607	365.17667	243.78687	183.09197	Q	3661.50984	1831.25856	1221.17480	916.13292	28
7	844.37301	422.69014	282.12919	211.84871	D	3533.45127	1767.22927	1178.48861	884.11827	27
8	931.40504	466.20616	311.13986	233.60672	S	3418.42432	1709.71580	1140.14629	855.36154	26
9	1045.44797	523.22762	349.15417	262.11745	N	3331.39229	1666.19979	1111.13562	833.60353	25
10	1142.50073	571.75400	381.50509	286.38064	P	3217.34937	1609.17832	1073.12131	805.09280	24
11	1302.53138	651.76933	434.84864	326.38830	C-Carbami...	3120.29660	1560.65194	1040.77039	780.82961	23
12	1415.61544	708.31136	472.54333	354.65932	L	2960.26595	1480.63662	987.42684	740.82195	22
13	1502.64747	751.82737	501.55401	376.41733	S	2847.18189	1424.09458	949.73215	712.55093	21
14	1749.75306	875.38017	583.92254	438.19372	T-Fuc(1)	2760.14986	1380.57857	920.72147	690.79292	20
15	1846.80582	923.90655	616.27346	462.45691	P	2513.04427	1257.02578	838.35294	629.01653	19
16	2006.83647	1003.92187	669.61701	502.46458	C-Carbami...	2415.99151	1208.49939	806.00202	604.75334	18
17	2134.93143	1067.96936	712.31533	534.48832	K	2255.96086	1128.48407	752.65847	564.74567	17
18	2248.97436	1124.99082	750.32964	562.99905	N	2127.86590	1064.43659	709.96015	532.72193	16
19	2320.01148	1160.50938	774.00868	580.75833	A	2013.82297	1007.41512	671.94584	504.21120	15
20	2377.03294	1189.02011	793.01583	595.01369	G	1942.78586	971.89657	648.26680	486.45192	14
21	2904.21796	1452.61262	968.74417	726.80995	T-Hex(1)P...	1885.76439	943.38584	629.25965	472.19656	13
22	3064.24861	1532.62794	1022.08772	766.81761	C-Carbami...	1358.57938	679.79333	453.53131	340.40030	12
23	3201.30752	1601.15740	1067.77402	801.08234	H	1198.54873	599.77800	400.18776	300.39264	11
24	3300.37593	1650.69160	1100.79683	825.84944	V	1061.48981	531.24855	354.50146	266.12791	10
25	3399.44435	1700.22581	1133.81963	850.61654	V	962.42140	481.71434	321.47865	241.36081	9
26	3514.47129	1757.73928	1172.16195	879.37328	D	863.35299	432.18013	288.45585	216.59370	8
27	3651.53020	1826.26874	1217.84825	913.63801	H	748.32604	374.66666	250.11353	187.83697	7
28	3708.55166	1854.77947	1236.85541	927.89337	G	611.26713	306.13720	204.42723	153.57224	6
29	3765.57313	1883.29020	1255.86256	942.14874	G	554.24567	277.62647	185.42007	139.31687	5
30	3866.62081	1933.81404	1289.54512	967.41066	T	497.22420	249.11574	166.41292	125.06151	4
31	3965.68922	1983.34825	1322.56792	992.17776	V	396.17653	198.59190	132.73036	99.79959	3
32	4080.71616	2040.86172	1360.91024	1020.93450	D	297.10811	149.05769	99.70756	75.03249	2
33					Y	182.08117	91.54422	61.36524	46.27575	1

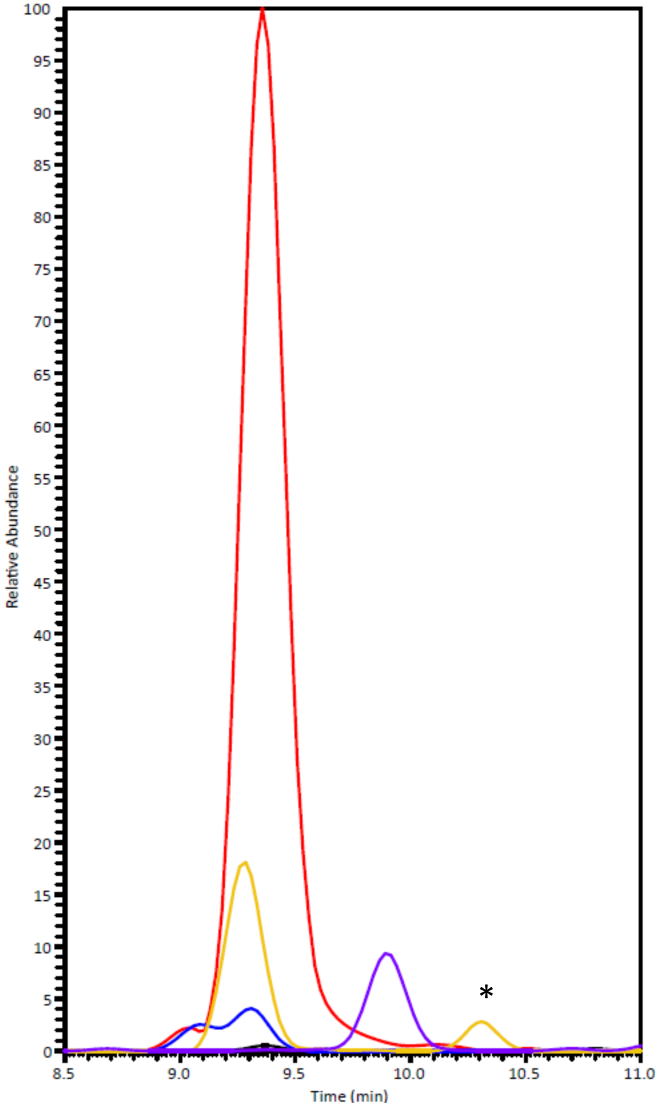

FP040621_17.raw #3571 RT: 9.3178 min
FTMS, 1066.7068@hcd27.00, z=+4, Mono m/z=1066.20508 Da, MH+=4261.79848 Da, Match Tol.=20 ppm

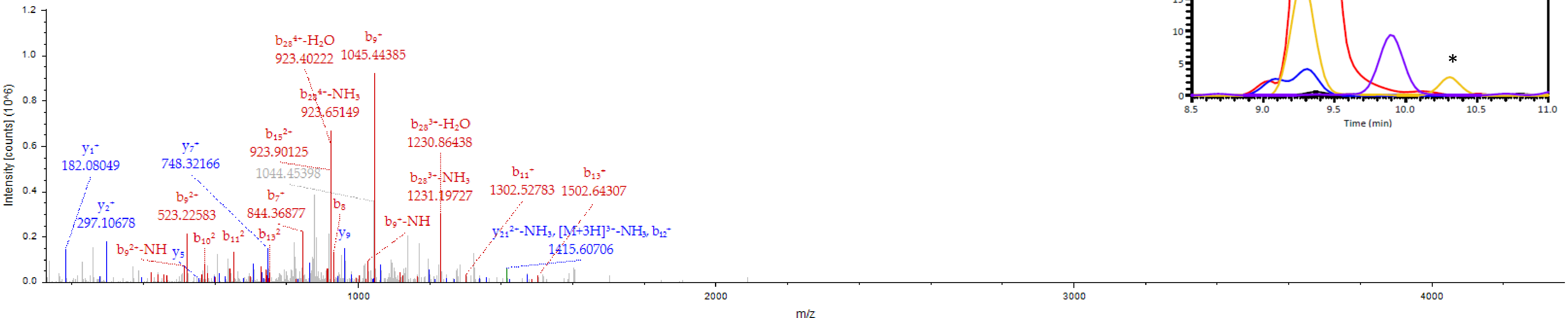

Figure S7B

EGF 3 : 113NGGTCDLLTLTEYK126

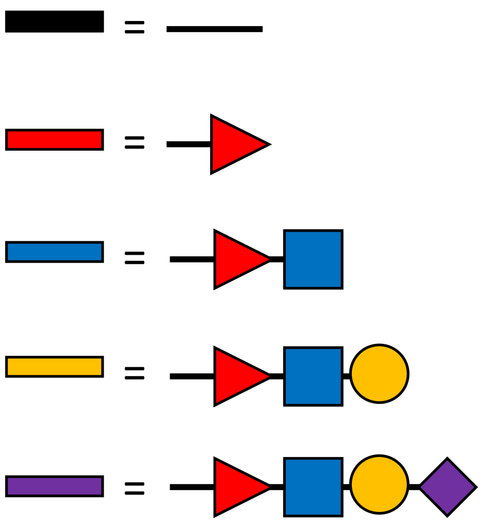

#1	b+	b2+	Seq.	y+	y2+	#2
1	115.05020	58.02874	N			14
2	172.07167	86.53947	G	1616.77238	808.88983	13
3	229.09313	115.05020	G	1559.75092	780.37910	12
4	476.19872	238.60300	T-Fuc(1)	1502.72945	751.86836	11
5	636.22937	318.61832	C-Carbami...	1255.62387	628.31557	10
6	751.25631	376.13179	D	1095.59322	548.30025	9
7	864.34037	432.67383	L	980.56627	490.78678	8
8	977.42444	489.21586	L	867.48221	434.24474	7
9	1078.47212	539.73970	T	754.39815	377.70271	6
10	1191.55618	596.28173	L	653.35047	327.17887	5
11	1292.60386	646.80557	T	540.26640	270.63684	4
12	1421.64645	711.32686	E	439.21873	220.11300	3
13	1584.70978	792.85853	Y	310.17613	155.59170	2
14			K	147.11280	74.06004	1

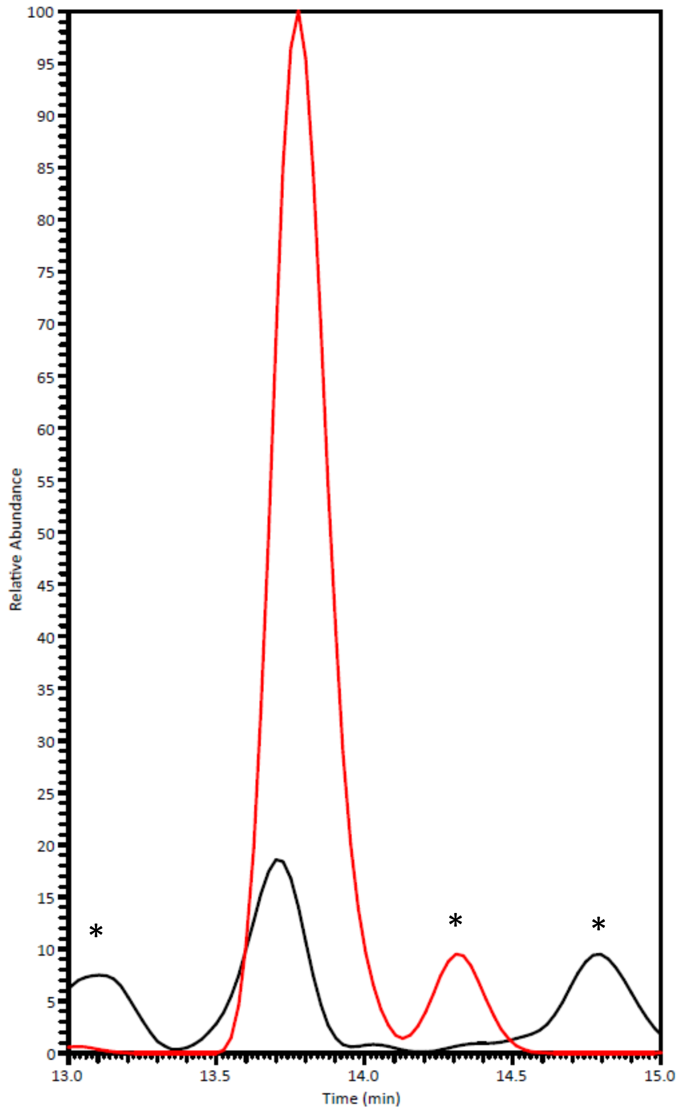

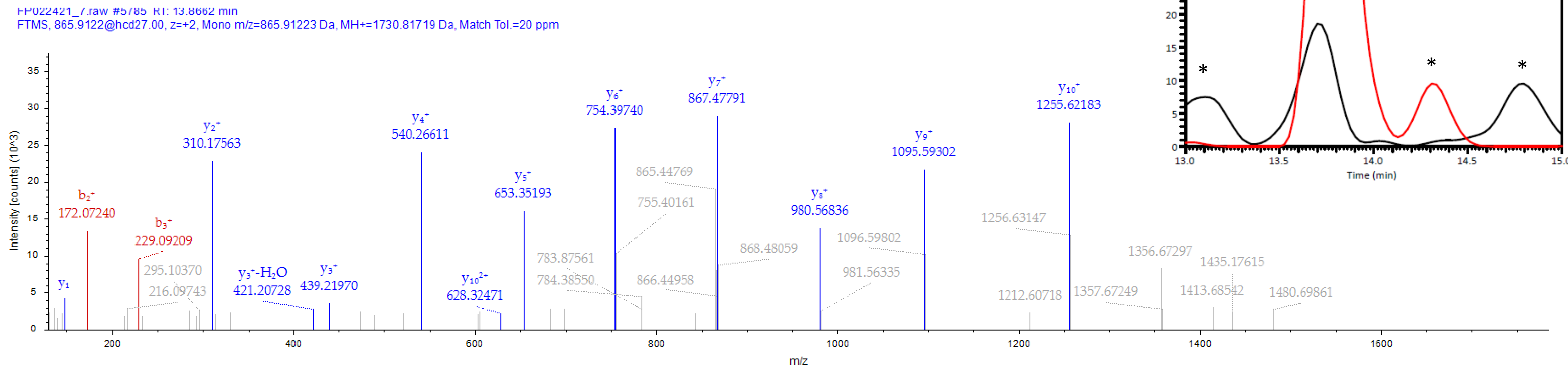

Figure S7C

EGF 5 : ^{163}ICRCPPGFHGPTCRQDVNECSQNPGLCRHGGTCHNEIGSY^{202}

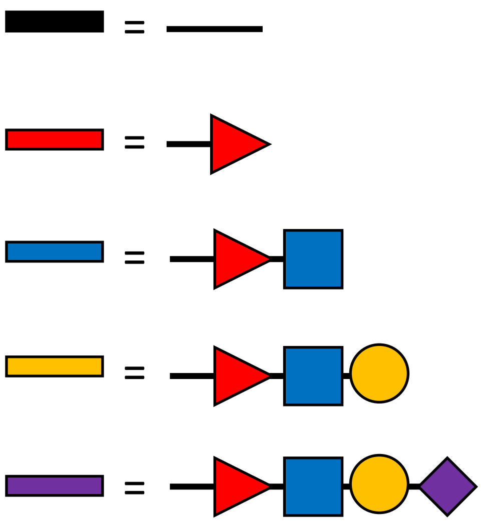

#1	b⁺	b²⁺	b³⁺	b⁴⁺	b⁵⁺	b⁶⁺	Seq.	y⁺	y²⁺	y³⁺	y⁴⁺	y⁵⁺	y⁶⁺	#2
1	114.09134	57.54931	38.70196	29.27829	23.62409	19.85462	I							40
2	274.12199	137.56463	92.04551	69.28595	55.63022	46.52640	C-Carbami...	4717.96531	2359.48629	1573.32662	1180.24679	944.39888	787.16695	39
3	430.22310	215.61519	144.07922	108.31123	86.85044	72.54325	R	4557.93466	2279.47097	1519.98307	1140.23912	912.39275	760.49517	38
4	590.25375	295.63051	197.42277	148.31889	118.85657	99.21502	C-Carbami...	4401.83355	2201.42041	1467.94937	1101.21385	881.17253	734.47832	37
5	687.30651	344.15689	229.77369	172.58209	138.26712	115.39048	P	4241.80290	2121.40509	1414.60582	1061.20618	849.16640	707.80655	36
6	784.35928	392.68328	262.12461	196.84528	157.67768	131.56594	P	4144.75014	2072.87871	1382.25490	1036.94299	829.75585	691.63109	35
7	841.38074	421.19401	281.13176	211.10064	169.08197	141.06952	G	4047.69737	2024.35233	1349.90398	1012.67980	810.34530	675.45563	34
8	988.44915	494.72822	330.15457	247.86775	198.49565	165.58092	F	3990.67591	1995.84159	1330.89682	998.42444	798.94100	665.95205	33
9	1125.50807	563.25767	375.84087	282.13247	225.90743	188.42407	H	3843.60750	1922.30739	1281.87402	961.65733	769.52732	641.44065	32
10	1182.52953	591.76840	394.84803	296.38784	237.31173	197.92765	G	3706.54859	1853.77793	1236.18771	927.39260	742.11554	618.59749	31
11	1279.58229	640.29478	427.19895	320.65103	256.72228	214.10311	P	3649.52712	1825.26720	1217.18056	913.13724	730.71125	609.09392	30
12	1526.68788	763.84758	509.56748	382.42743	306.14340	255.28738	T-Fuc(1)	3552.47436	1776.74082	1184.82964	888.87405	711.30069	592.91846	29
13	1686.71853	843.86290	562.91103	422.43509	338.14953	281.95915	C-Carbami...	3305.36877	1653.18802	1102.46111	827.09765	661.87958	551.73419	28
14	1842.81964	921.91346	614.94473	461.46037	369.36975	307.97600	R	3145.33812	1573.17270	1049.11756	787.08999	629.87345	525.06242	27
15	1970.87822	985.94275	657.63092	493.47501	394.98146	329.31910	Q	2989.23701	1495.12214	997.08385	748.06471	598.65322	499.04557	26
16	2085.90516	1043.45622	695.97324	522.23175	417.98685	348.49026	D	2861.17843	1431.09286	954.39766	716.05007	573.04151	477.70247	25
17	2184.97357	1092.99043	728.99604	546.99885	437.80054	365.00166	V	2746.15149	1373.57938	916.05535	687.29333	550.03612	458.53131	24
18	2299.01650	1150.01189	767.01035	575.50958	460.60912	384.00881	N	2647.08308	1324.04518	883.03254	662.52623	530.22244	442.01991	23
19	2428.05909	1214.53319	810.02455	607.77023	486.41764	405.51591	E	2533.04015	1267.02371	845.01823	634.01549	507.41385	423.01276	22
20	2588.08974	1294.54851	863.36810	647.77789	518.42377	432.18769	C-Carbami...	2403.99756	1202.50242	802.00404	601.75485	481.60533	401.50566	21
21	2675.12177	1338.06452	892.37877	669.53590	535.83018	446.69303	S	2243.96691	1122.48709	748.66049	561.74718	449.59920	374.83388	20
22	2803.18035	1402.09381	935.06497	701.55054	561.44189	468.03612	Q	2156.93488	1078.97108	719.64981	539.98918	432.19280	360.32854	19
23	2917.22328	1459.11528	973.07928	730.06128	584.25048	487.04328	N	2028.87630	1014.94179	676.96362	507.97453	406.58108	338.98545	18
24	3014.27604	1507.64166	1005.43020	754.32447	603.66103	503.21874	P	1914.83337	957.92033	638.94931	479.46380	383.77250	319.97829	17
25	3071.29750	1536.15239	1024.43735	768.57983	615.06532	512.72231	G	1817.78061	909.39394	606.59839	455.20061	364.36194	303.80283	16
26	3184.38157	1592.69442	1062.13204	796.85085	637.68213	531.56966	L	1760.75915	880.88321	587.59123	440.94524	352.95765	294.29926	15
27	3344.41222	1672.70975	1115.47559	836.85851	669.68826	558.24143	C-Carbami...	1647.67508	824.34118	549.89655	412.67423	330.34084	275.45191	14
28	3500.51333	1750.76030	1167.50929	875.88379	700.90849	584.25828	R	1487.64443	744.32586	496.55300	372.66657	298.33471	248.78014	13
29	3637.57224	1819.28976	1213.19560	910.14852	728.32027	607.10144	H	1331.54332	666.27530	444.51929	333.64129	267.11449	222.76328	12
30	3694.59370	1847.80049	1232.20275	924.40388	739.72456	616.60501	G	1194.48441	597.74584	398.83299	299.37656	239.70270	199.92013	11
31	3751.61517	1876.31122	1251.20991	938.65925	751.12885	626.10859	G	1137.46295	569.23511	379.82583	285.12119	228.29841	190.41656	10
32	3852.66284	1926.83506	1284.89247	963.92117	771.33839	642.94987	T	1080.44148	540.72438	360.81868	270.86583	216.89412	180.91298	9
33	4012.69349	2006.85038	1338.23602	1003.92883	803.34452	669.62165	C-Carbami...	979.39381	490.20054	327.13612	245.60391	196.68458	164.07170	8
34	4149.75240	2075.37984	1383.92232	1038.19356	830.75630	692.46480	H	819.36316	410.18522	273.79257	205.59625	164.67845	137.39992	7
35	4263.79533	2132.40130	1421.93663	1066.70429	853.56489	711.47195	N	682.30425	341.65576	228.10627	171.33152	137.26667	114.55677	6
36	4392.83792	2196.92260	1464.95083	1098.96494	879.37341	732.97905	E	568.26132	284.63430	190.09196	142.82079	114.45809	95.54962	5
37	4505.92199	2253.46463	1502.64551	1127.23595	901.99022	751.82640	I	439.21873	220.11300	147.07776	110.56014	88.64957	74.04252	4
38	4562.94345	2281.97536	1521.65267	1141.49132	913.39451	761.32997	G	326.13466	163.57097	109.38307	82.28912	66.03275	55.19517	3
39	4649.97548	2325.49138	1550.66334	1163.24933	930.80092	775.83531	S	269.11320	135.06024	90.37592	68.03376	54.62846	45.69160	2
40							Y	182.08117	91.54422	61.36524	46.27575	37.22206	31.18626	1

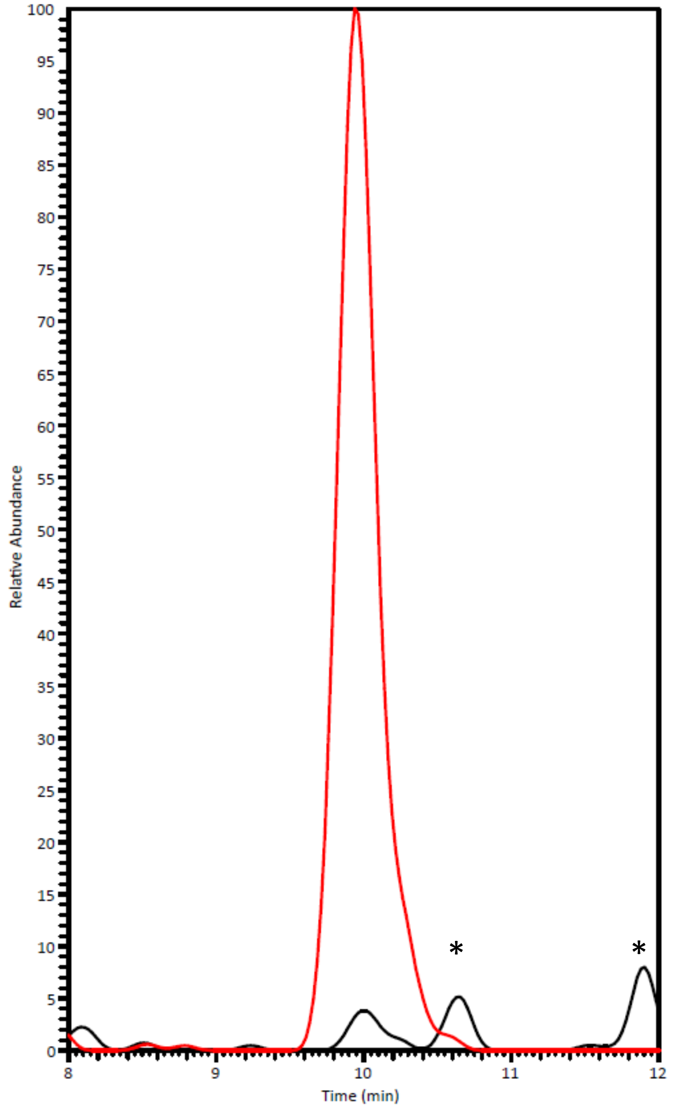

FP040621_17.raw #3822 RT: 9.8943 min
FTMS, 806.3533@hcd27.00, z=+6, Mono m/z=806.01978 Da, MH+=4831.08227 Da, Match Tol.=20 ppm

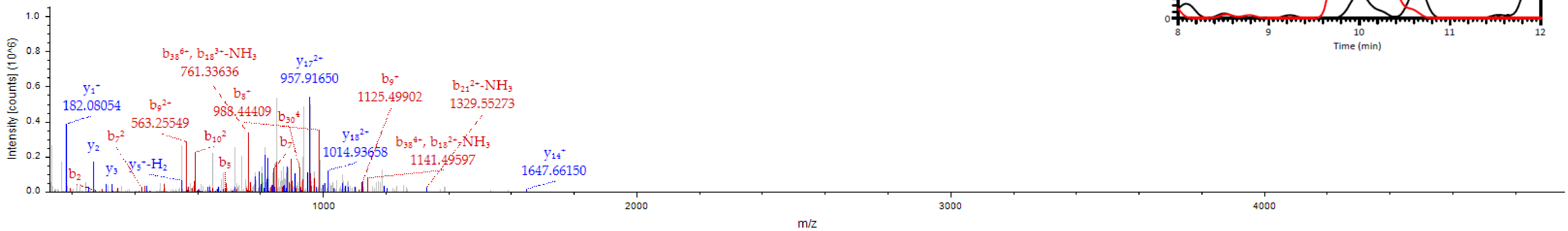

Figure S7D

EGF 6 : ^{199}IGSYRCACRATHTGPHCELPYVPCPSPCQNGGTCRPTGDTTHE^{242}

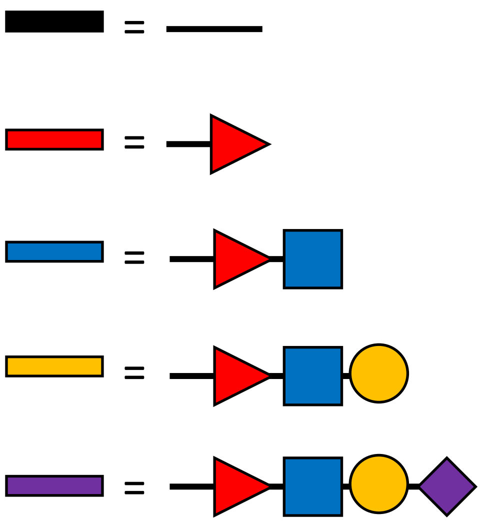

#1	b⁺	b²⁺	b³⁺	b⁴⁺	b⁵⁺	Seq.	y⁺	y²⁺	y³⁺	y⁴⁺	y⁵⁺	#2
1	114.09134	57.54931	38.70196	29.27829	23.62409	I						44
2	171.11280	86.06004	57.70912	43.53366	35.02838	G	5036.09678	2518.55203	1679.37044	1259.77965	1008.02518	43
3	258.14483	129.57605	86.71980	65.29167	52.43479	S	4979.07531	2490.04130	1660.36329	1245.52429	996.62088	42
4	421.20816	211.10772	141.07424	106.05750	85.04745	Y	4892.04329	2446.52528	1631.35261	1223.76628	979.21448	41
5	577.30927	289.15827	193.10794	145.08278	116.26768	R	4728.97996	2364.99362	1576.99817	1183.00045	946.60181	40
6	737.33992	369.17360	246.45149	185.09044	148.27381	C-Carbami...	4572.87885	2286.94306	1524.96447	1143.97517	915.38159	39
7	808.37703	404.69216	270.13053	202.84972	162.48123	A	4412.84820	2206.92774	1471.62092	1103.96751	883.37546	38
8	968.40768	484.70748	323.47408	242.85738	194.48736	C-Carbami...	4341.81108	2171.40918	1447.94188	1086.20823	869.16804	37
9	1124.50879	562.75804	375.50778	281.88266	225.70758	R	4181.78044	2091.39386	1394.59833	1046.20057	837.16191	36
10	1195.54591	598.27659	399.18682	299.64193	239.91500	A	4025.67932	2013.34330	1342.56463	1007.17529	805.94169	35
11	1296.59359	648.80043	432.86938	324.90385	260.12454	T	3954.64221	1977.82474	1318.88559	989.41601	791.73426	34
12	1433.65250	717.32989	478.55568	359.16858	287.53632	H	3853.59453	1927.30090	1285.20303	964.15409	771.52473	33
13	1534.70018	767.85373	512.23824	384.43050	307.74586	T	3716.53562	1858.77145	1239.51672	929.88936	744.11295	32
14	1591.72164	796.36446	531.24540	398.68587	319.15015	G	3615.48794	1808.24761	1205.83417	904.62744	723.90341	31
15	1688.77440	844.89084	563.59632	422.94906	338.56070	P	3558.46648	1779.73688	1186.82701	890.37208	712.49912	30
16	1825.83332	913.42030	609.28262	457.21379	365.97248	H	3461.41372	1731.21050	1154.47609	866.10889	693.08856	29
17	1985.86396	993.43562	662.62617	497.22145	397.97861	C-Carbami...	3324.35480	1662.68104	1108.78979	831.84416	665.67678	28
18	2114.90656	1057.95692	705.64037	529.48210	423.78713	E	3164.32415	1582.66572	1055.44624	791.83650	633.67065	27
19	2227.99062	1114.49895	743.33506	557.75311	446.40395	L	3035.28156	1518.14442	1012.43204	759.57585	607.86213	26
20	2325.04338	1163.02533	775.68598	582.01630	465.81450	P	2922.19750	1461.60239	974.73735	731.30483	585.24532	25
21	2488.10671	1244.55699	830.04042	622.78214	498.42716	Y	2825.14473	1413.07601	942.38643	707.04164	565.83477	24
22	2587.17513	1294.09120	863.06323	647.54924	518.24085	V	2662.08141	1331.54434	888.03199	666.27581	533.22210	23
23	2684.22789	1342.61758	895.41415	671.81243	537.65140	P	2563.01299	1282.01013	855.00918	641.50871	513.40842	22
24	2844.25854	1422.63291	948.75770	711.82009	569.65753	C-Carbami...	2465.96023	1233.48375	822.65826	617.24551	493.99787	21
25	2931.29057	1466.14892	977.76837	733.57810	587.06393	S	2305.92958	1153.46843	769.31471	577.23785	461.99174	20
26	3028.34333	1514.67530	1010.11929	757.84129	606.47449	P	2218.89755	1109.95241	740.30403	555.47985	444.58533	19
27	3115.37536	1558.19132	1039.12997	779.59930	623.88089	S	2121.84479	1061.42603	707.95311	531.21665	425.17478	18
28	3212.42812	1606.71770	1071.48089	803.86249	643.29145	P	2034.81276	1017.91002	678.94244	509.45865	407.76837	17
29	3372.45877	1686.73302	1124.82444	843.87015	675.29758	C-Carbami...	1937.76000	969.38364	646.59152	485.19546	388.35782	16
30	3500.51735	1750.76231	1167.51063	875.88479	700.90929	Q	1777.72935	889.36831	593.24797	445.18779	356.35169	15
31	3615.54429	1808.27578	1205.85295	904.64153	723.91468	N-Deamid...	1649.67077	825.33902	550.56177	413.17315	330.73998	14
32	3672.56576	1836.78652	1224.86010	918.89690	735.31897	G	1534.64383	767.82555	512.21946	384.41641	307.73459	13
33	3729.58722	1865.29725	1243.86726	933.15226	746.72327	G	1477.62236	739.31482	493.21231	370.16105	296.33029	12
34	3976.69281	1988.85004	1326.23579	994.92866	796.14438	T-Fuc(1)	1420.60090	710.80409	474.20515	355.90568	284.92600	11
35	4136.72346	2068.86537	1379.57934	1034.93632	828.15051	C-Carbami...	1173.49531	587.25129	391.83662	294.12929	235.50488	10
36	4292.82457	2146.91592	1431.61304	1073.96160	859.37073	R	1013.46466	507.23597	338.49307	254.12162	203.49875	9
37	4389.87733	2195.44230	1463.96396	1098.22479	878.78129	P	857.36355	429.18541	286.45937	215.09635	172.27853	8
38	4490.92501	2245.96614	1497.64652	1123.48671	898.99082	T	760.31079	380.65903	254.10845	190.83315	152.86798	7
39	4547.94647	2274.47687	1516.65368	1137.74208	910.39512	G	659.26311	330.13519	220.42589	165.57123	132.65844	6
40	4662.97342	2331.99035	1554.99599	1166.49881	933.40050	D	602.24165	301.62446	201.41873	151.31587	121.25415	5
41	4764.02109	2382.51419	1588.67855	1191.76073	953.61004	T	487.21470	244.11099	163.07642	122.55913	98.24876	4
42	4865.06877	2433.03802	1622.36111	1217.02265	973.81958	T	386.16702	193.58715	129.39386	97.29721	78.03923	3
43	5002.12768	2501.56748	1668.04741	1251.28738	1001.23136	H	285.11935	143.06331	95.71130	72.03529	57.82969	2
44						E	148.06043	74.53386	50.02500	37.77057	30.41791	1

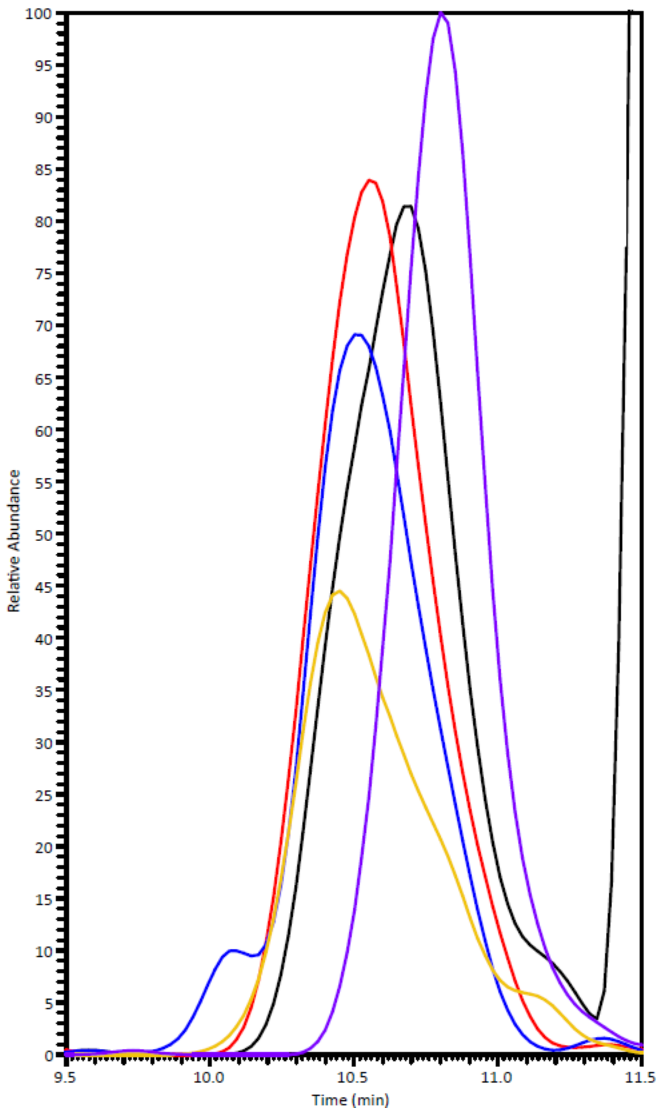

FP040621_14.raw #4039 RT: 10.5590 min
FTMS, 1031.0482@hcd27.00, z=+5, Mono m/z=1030.64575 Da, MH+=5149.19965 Da, Match Tol.=20 ppm

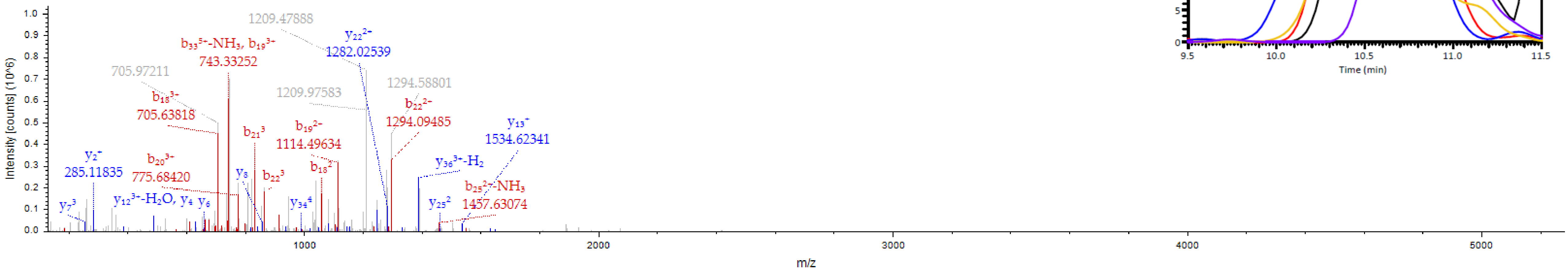

Figure S7E

EGF 8 : [280]NCRCPPEWTGQYCTEDVDECQLMPNACQNGGTCHNTHGGY[319]

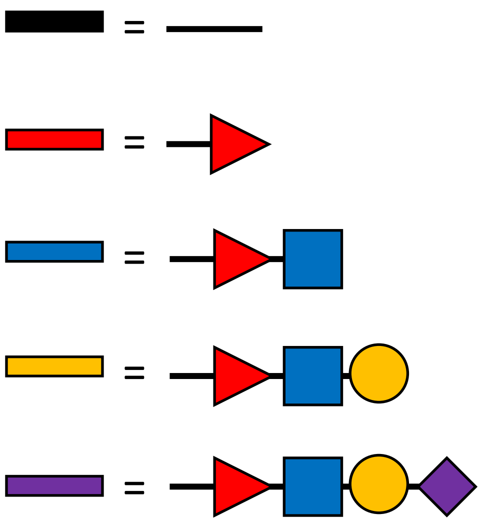

#1	b⁺	b²⁺	b³⁺	b⁴⁺	Seq.	y⁺	y²⁺	y³⁺	y⁴⁺	#2
1	115.05020	58.02874	39.02159	29.51801	N					40
2	275.08085	138.04406	92.36514	69.52567	C-Carbami...	5008.93650	2504.97189	1670.31702	1252.98958	39
3	431.18196	216.09462	144.39884	108.55095	R	4848.90585	2424.95656	1616.97347	1212.98192	38
4	591.21261	296.10994	197.74239	148.55861	C-Carbami...	4692.80474	2346.90601	1564.93976	1173.95664	37
5	688.26538	344.63633	230.09331	172.82180	P	4532.77409	2266.89068	1511.59621	1133.94898	36
6	785.31814	393.16271	262.44423	197.08499	P	4435.72133	2218.36430	1479.24529	1109.68579	35
7	914.36073	457.68400	305.45843	229.34564	E	4338.66856	2169.83792	1446.89437	1085.42260	34
8	1100.44005	550.72366	367.48487	275.86547	W	4209.62597	2105.31662	1403.88017	1053.16195	33
9	1201.48772	601.24750	401.16743	301.12739	T	4023.54666	2012.27697	1341.85374	1006.64212	32
10	1258.50919	629.75823	420.17458	315.38275	G	3922.49898	1961.75313	1308.17118	981.38020	31
11	1386.56776	693.78752	462.86077	347.39740	Q	3865.47751	1933.24240	1289.16402	967.12484	30
12	1549.63109	775.31919	517.21522	388.16323	Y	3737.41894	1869.21311	1246.47783	935.11019	29
13	1709.66174	855.33451	570.55877	428.17089	C-Carbami...	3574.35561	1787.68144	1192.12339	894.34436	28
14	2159.84670	1080.42699	720.62042	540.71713	T-HexNAc(...	3414.32496	1707.66612	1138.77984	854.33670	27
15	2288.88929	1144.94829	763.63462	572.97778	E	2964.14000	1482.57364	988.71818	741.79046	26
16	2403.91624	1202.46176	801.97693	601.73452	D	2835.09741	1418.05234	945.70399	709.52981	25
17	2502.98465	1251.99596	834.99973	626.50162	V	2720.07046	1360.53887	907.36167	680.77307	24
18	2618.01159	1309.50944	873.34205	655.25836	D	2621.00205	1311.00466	874.33887	656.00597	23
19	2747.05419	1374.03073	916.35625	687.51900	E	2505.97511	1253.49119	835.99655	627.24923	22
20	2907.08484	1454.04606	969.69980	727.52667	C-Carbami...	2376.93251	1188.96990	792.98236	594.98859	21
21	3035.14341	1518.07535	1012.38599	759.54131	Q	2216.90186	1108.95457	739.63881	554.98092	20
22	3148.22748	1574.61738	1050.08068	787.81233	L	2088.84329	1044.92528	696.95261	522.96628	19
23	3279.26796	1640.13762	1093.76084	820.57245	M	1975.75922	988.38325	659.25793	494.69526	18
24	3376.32073	1688.66400	1126.11176	844.83564	P	1844.71874	922.86301	615.57776	461.93514	17
25	3490.36365	1745.68546	1164.12607	873.34637	N	1747.66598	874.33663	583.22684	437.67195	16
26	3561.40077	1781.20402	1187.80511	891.10565	A	1633.62305	817.31516	545.21253	409.16122	15
27	3721.43142	1861.21935	1241.14866	931.11331	C-Carbami...	1562.58593	781.79661	521.53350	391.40194	14
28	3849.48999	1925.24863	1283.83485	963.12796	Q	1402.55529	701.78128	468.18995	351.39428	13
29	3963.53292	1982.27010	1321.84916	991.63869	N	1274.49671	637.75199	425.50375	319.37963	12
30	4020.55438	2010.78083	1340.85631	1005.89405	G	1160.45378	580.73053	387.48944	290.86890	11
31	4077.57585	2039.29156	1359.86347	1020.14942	G	1103.43232	552.21980	368.48229	276.61354	10
32	4178.62353	2089.81540	1393.54603	1045.41134	T	1046.41085	523.70907	349.47514	262.35817	9
33	4338.65417	2169.83073	1446.88958	1085.41900	C-Carbami...	945.36318	473.18523	315.79258	237.09625	8
34	4475.71309	2238.36018	1492.57588	1119.68373	H	785.33253	393.16990	262.44903	197.08859	7
35	4589.75601	2295.38165	1530.59019	1148.19446	N	648.27361	324.64045	216.76272	162.82386	6
36	4690.80369	2345.90548	1564.27275	1173.45638	T	534.23069	267.61898	178.74841	134.31313	5
37	4827.86260	2414.43494	1609.95905	1207.72111	H	433.18301	217.09514	145.06585	109.05121	4
38	4884.88407	2442.94567	1628.96621	1221.97647	G	296.12410	148.56569	99.37955	74.78648	3
39	4941.90553	2471.45640	1647.97336	1236.23184	G	239.10263	120.05496	80.37240	60.53112	2
40					Y	182.08117	91.54422	61.36524	46.27575	1

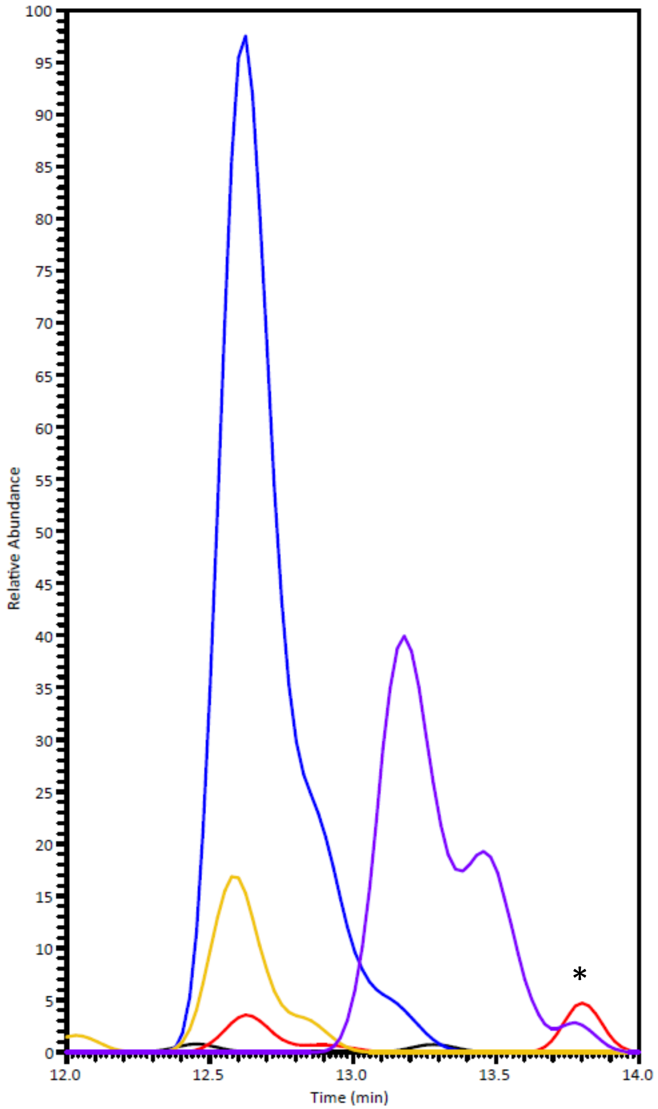

FP040621_17.raw #5016 RT: 12.5926 min
FTMS, 1282.2562@hcd27.00, z=+4, Mono m/z=1281.51367 Da, MH+=5123.03286 Da, Match Tol.=20 ppm

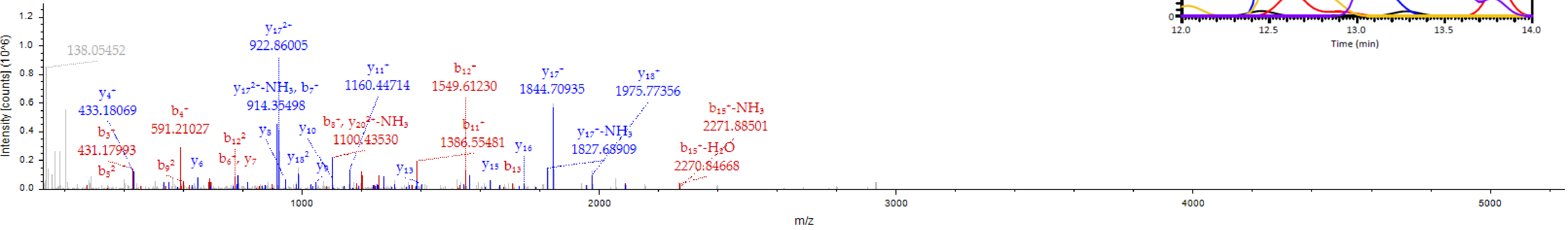

Figure S7F

EGF 9 : 346QGATCHDRVASFY358

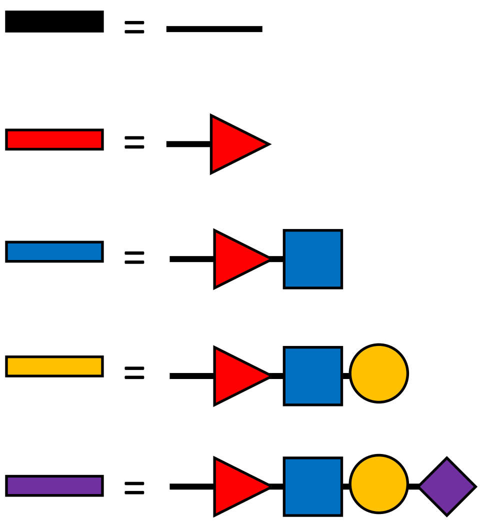

#1	b+	b2+	Seq.	y+	y2+	#2
1	129.06585	65.03657	Q			13
2	186.08732	93.54730	G	1529.66892	765.33810	12
3	257.12443	129.06585	A	1472.64745	736.82737	11
4	358.17211	179.58969	T	1401.61034	701.30881	10
5	518.20276	259.60502	C-Carbami...	1300.56266	650.78497	9
6	655.26167	328.13447	H	1140.53201	570.76965	8
7	770.28861	385.64794	D	1003.47310	502.24019	7
8	926.38972	463.69850	R	888.44616	444.72672	6
9	1025.45814	513.23271	V	732.34505	366.67616	5
10	1096.49525	548.75126	A	633.27663	317.14196	4
11	1329.58519	665.29623	S-Fuc(1)	562.23952	281.62340	3
12	1476.65360	738.83044	F	329.14958	165.07843	2
13			Y	182.08117	91.54422	1

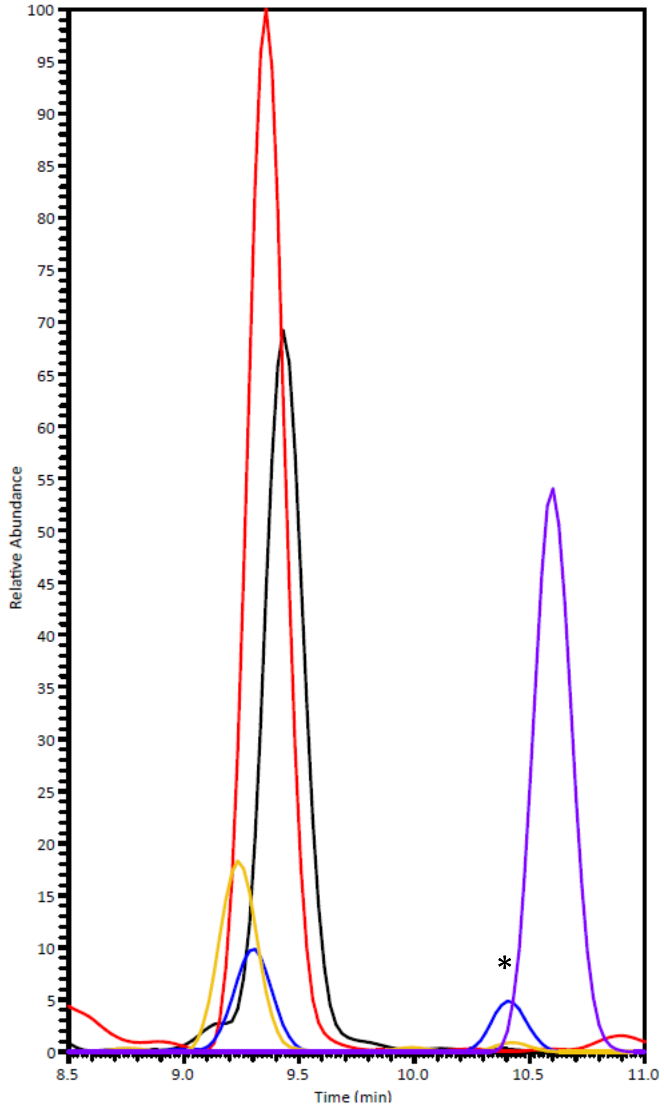

FP040621_17.raw #3584 RT: 9.3448 min
FTMS, 829.3718@hcd27.00, z=+2, Mono m/z=829.37177 Da, MH+=1657.73625 Da, Match Tol.=20 ppm

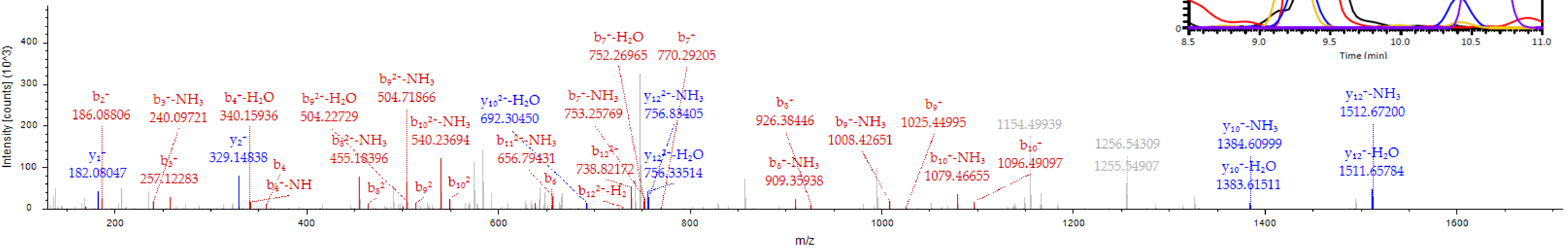

Figure S7G

EGF 12 : 451IDVNECISNPCQNDATCLDQIGE473

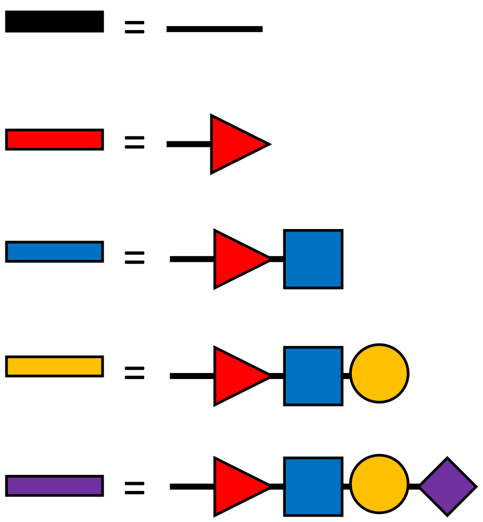

#1	b^+	b^{2+}	b^{3+}	Seq.	y^+	y^{2+}	y^{3+}	#2
1	114.09134	57.54931	38.70196	I				23
2	229.11828	115.06278	77.04428	D	3124.22864	1562.61796	1042.08107	22
3	328.18670	164.59699	110.06708	V	3009.20170	1505.10449	1003.73875	21
4	442.22962	221.61845	148.08139	N	2910.13329	1455.57028	970.71595	20
5	571.27222	286.13975	191.09559	E	2796.09036	1398.54882	932.70164	19
6	731.30287	366.15507	244.43914	C-Carbami...	2667.04776	1334.02752	889.68744	18
7	844.38693	422.69710	282.13383	I	2507.01712	1254.01220	836.34389	17
8	1357.55630	679.28179	453.19028	S-Hex(1)P...	2393.93305	1197.47016	798.64920	16
9	1471.59923	736.30325	491.20459	N	1880.76368	940.88548	627.59275	15
10	1568.65199	784.82963	523.55551	P	1766.72076	883.86402	589.57844	14
11	1728.68264	864.84496	576.89906	C-Carbami...	1669.66799	835.33763	557.22752	13
12	1856.74122	928.87425	619.58526	Q	1509.63734	755.32231	503.88397	12
13	1970.78414	985.89571	657.59957	N	1381.57877	691.29302	461.19777	11
14	2085.81109	1043.40918	695.94188	D	1267.53584	634.27156	423.18346	10
15	2156.84820	1078.92774	719.62092	A	1152.50890	576.75809	384.84115	9
16	2403.95379	1202.48053	801.98945	T-Fuc(1)	1081.47178	541.23953	361.16211	8
17	2563.98444	1282.49586	855.33300	C-Carbami...	834.36619	417.68674	278.79358	7
18	2677.06850	1339.03789	893.02768	L	674.33555	337.67141	225.45003	6
19	2792.09544	1396.55136	931.37000	D	561.25148	281.12938	187.75535	5
20	2920.15402	1460.58065	974.05619	Q	446.22454	223.61591	149.41303	4
21	3033.23808	1517.12268	1011.75088	I	318.16596	159.58662	106.72684	3
22	3090.25955	1545.63341	1030.75803	G	205.08190	103.04459	69.03215	2
23				E	148.06043	74.53386	50.02500	1

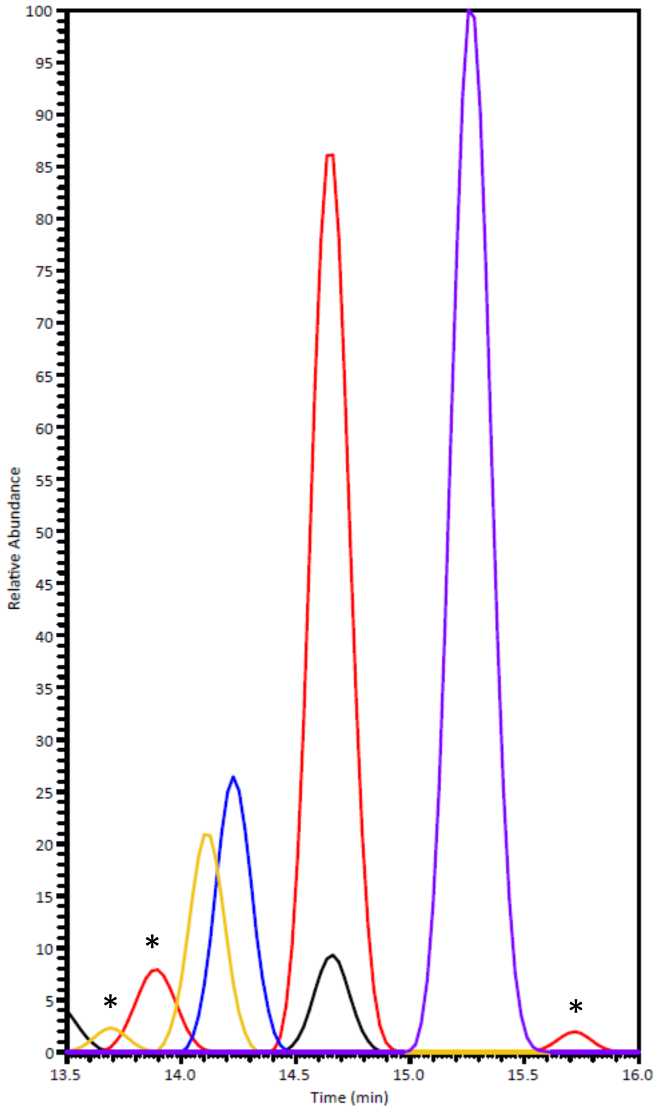

FP040621_14.raw #5781 RT: 14.6023 min
FTMS, 1080.1116@hcd27.00, z=+3, Mono m/z=1079.77917 Da, MH+=3237.32297 Da, Match Tol.=20 ppm

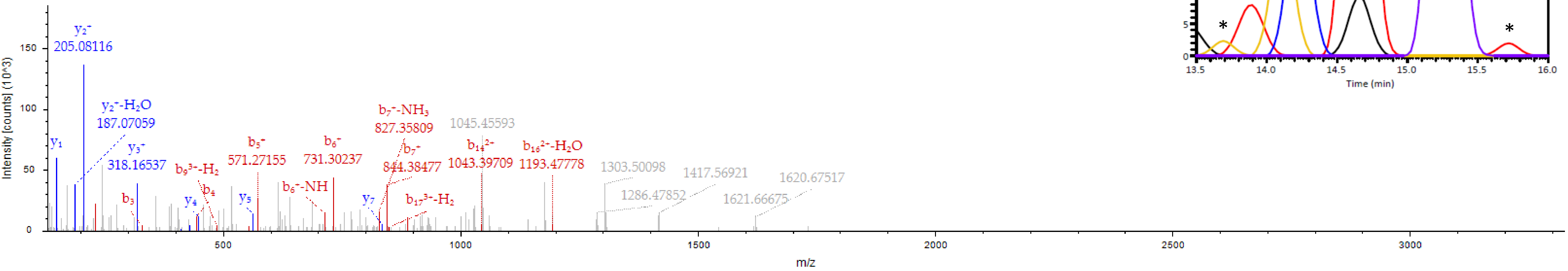

Figure S7H

EGF 16 : 614HGGTCQDRDNSYLCLCLK631

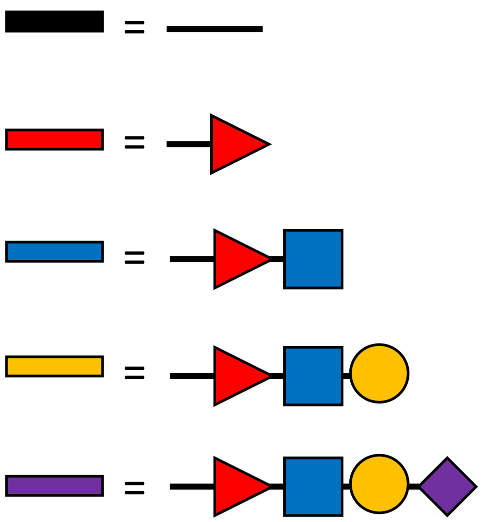

#1	b+	b2+	b3+	b4+	Seq.	y+	y2+	y3+	y4+	#2
1	138.06619	69.53673	46.69358	35.27200	H					18
2	195.08765	98.04746	65.70074	49.52737	G	2862.18493	1431.59610	954.73316	716.30169	17
3	252.10912	126.55820	84.70789	63.78274	G	2805.16346	1403.08537	935.72601	702.04632	16
4	1155.44232	578.22480	385.81896	289.61604	T-HexNAc(...	2748.14200	1374.57464	916.71885	687.79096	15
5	1315.47296	658.24012	439.16251	329.62370	C-Carbami...	1844.80880	922.90804	615.60778	461.95766	14
6	1443.53154	722.26941	481.84870	361.63834	Q	1684.77815	842.89271	562.26423	421.95000	13
7	1558.55848	779.78288	520.19101	390.39508	D	1556.71957	778.86343	519.57804	389.93535	12
8	1714.65960	857.83344	572.22472	429.42036	R	1441.69263	721.34995	481.23573	361.17862	11
9	1829.68654	915.34691	610.56703	458.17709	D	1285.59152	643.29940	429.20202	322.15334	10
10	1943.72947	972.36837	648.58134	486.68782	N	1170.56458	585.78593	390.85971	293.39660	9
11	2030.76149	1015.88439	677.59202	508.44583	S	1056.52165	528.76446	352.84540	264.88587	8
12	2193.82482	1097.41605	731.94646	549.21166	Y	969.48962	485.24845	323.83472	243.12786	7
13	2306.90889	1153.95808	769.64115	577.48268	L	806.42629	403.71678	269.48028	202.36203	6
14	2466.93954	1233.97341	822.98470	617.49034	C-Carbami...	693.34223	347.17475	231.78559	174.09101	5
15	2580.02360	1290.51544	860.67938	645.76136	L	533.31158	267.15943	178.44204	134.08335	4
16	2740.05425	1370.53076	914.02293	685.76902	C-Carbami...	420.22752	210.61740	140.74736	105.81234	3
17	2853.13831	1427.07279	951.71762	714.04004	L	260.19687	130.60207	87.40381	65.80467	2
18					K	147.11280	74.06004	49.70912	37.53366	1

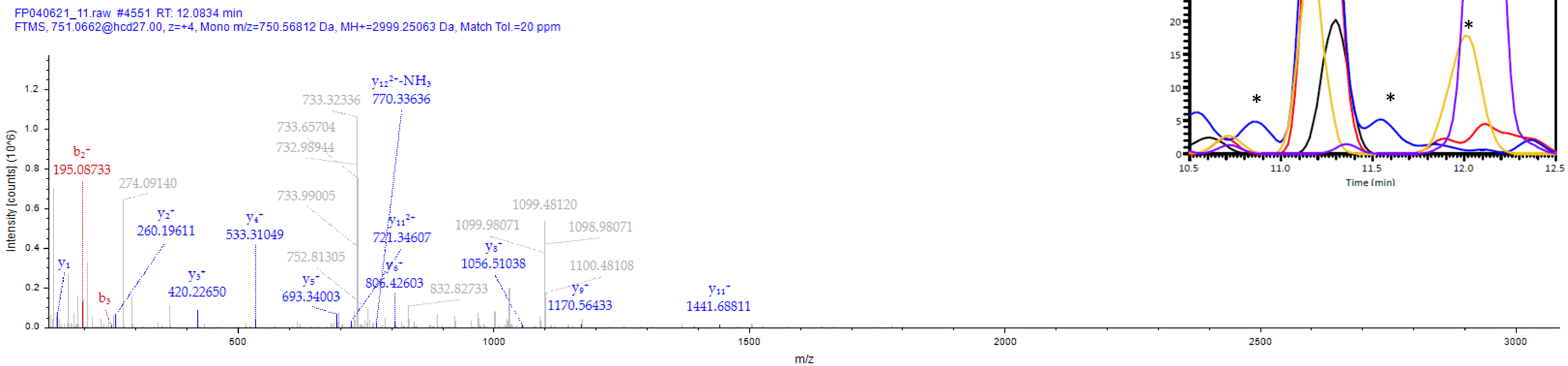

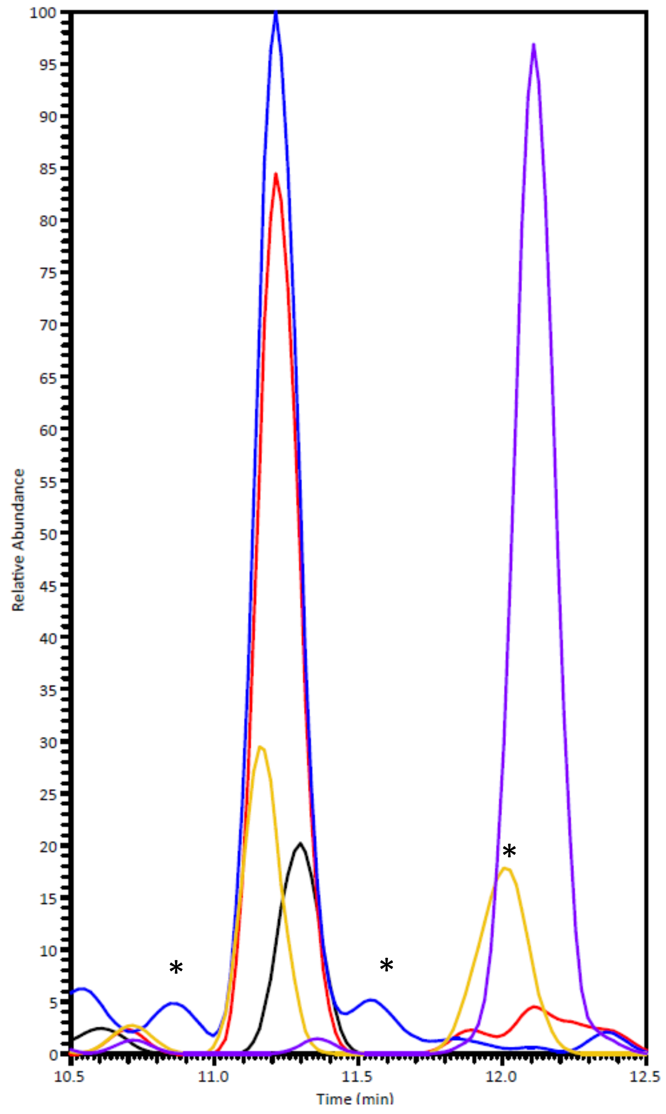

Figure S7I

EGF 18 : 682CAGSPCHNGGTCEDGIAGFTCRCPE706

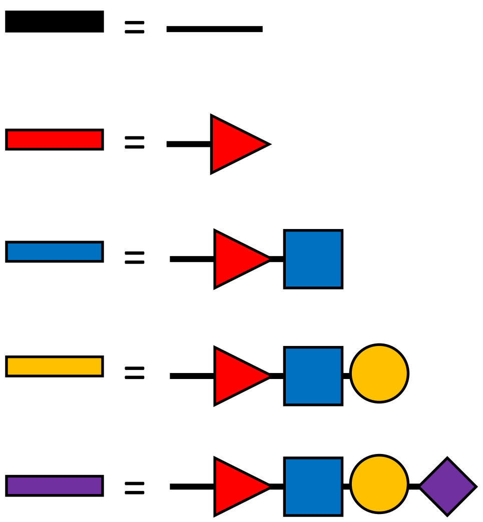

#1	b+	b2+	b3+	Seq.	y+	y2+	y3+	#2
1	161.03793	81.02260	54.35083	C-Carbami...				25
2	232.07504	116.54116	78.02986	A	2975.15465	1488.08096	992.38973	24
3	289.09650	145.05189	97.03702	G	2904.11753	1452.56241	968.71070	23
4	376.12853	188.56790	126.04769	S	2847.09607	1424.05167	949.70354	22
5	473.18129	237.09429	158.39862	P	2760.06404	1380.53566	920.69286	21
6	633.21194	317.10961	211.74217	C-Carbami...	2663.01128	1332.00928	888.34194	20
7	770.27085	385.63907	257.42847	H	2502.98063	1251.99395	834.99839	19
8	884.31378	442.66053	295.44278	N	2365.92172	1183.46450	789.31209	18
9	941.33525	471.17126	314.44993	G	2251.87879	1126.44303	751.29778	17
10	998.35671	499.68199	333.45709	G	2194.85733	1097.93230	732.29063	16
11	1448.54167	724.77447	483.51874	T-HexNAc(...	2137.83586	1069.42157	713.28347	15
12	1608.57232	804.78980	536.86229	C-Carbami...	1687.65090	844.32909	563.22182	14
13	1737.61491	869.31109	579.87649	E	1527.62025	764.31377	509.87827	13
14	1868.63677	934.82202	623.55044	D-Oxidation	1398.57766	699.79247	466.86407	12
15	1925.65823	963.33275	642.55760	G	1267.55580	634.28154	423.19012	11
16	2038.74230	1019.87479	680.25228	I	1210.53434	605.77081	404.18296	10
17	2109.77941	1055.39334	703.93132	A	1097.45028	549.22878	366.48828	9
18	2166.80087	1083.90408	722.93848	G	1026.41316	513.71022	342.80924	8
19	2313.86929	1157.43828	771.96128	F	969.39170	485.19949	323.80208	7
20	2414.91697	1207.96212	805.64384	T	822.32328	411.66528	274.77928	6
21	2574.94761	1287.97745	858.98739	C-Carbami...	721.27561	361.14144	241.09672	5
22	2731.04873	1366.02800	911.02109	R	561.24496	281.12612	187.75317	4
23	2891.07937	1446.04333	964.36464	C-Carbami...	405.14385	203.07556	135.71947	3
24	2988.13214	1494.56971	996.71556	P	245.11320	123.06024	82.37592	2
25				E	148.06043	74.53386	50.02500	1

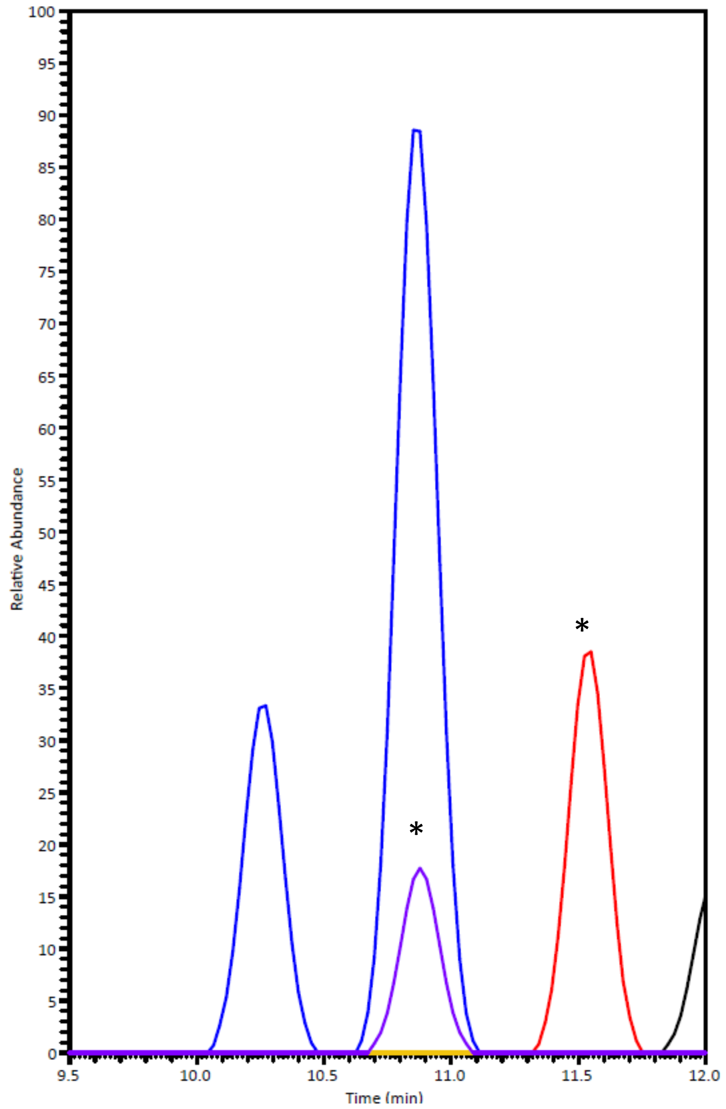

FP040621_14.raw #4185 RT: 10.8925 min
FTMS, 1046.0757@hcd27.00, z=+3, Mono m/z=1045.74023 Da, MH+=3135.20615 Da, Match Tol.=20 ppm

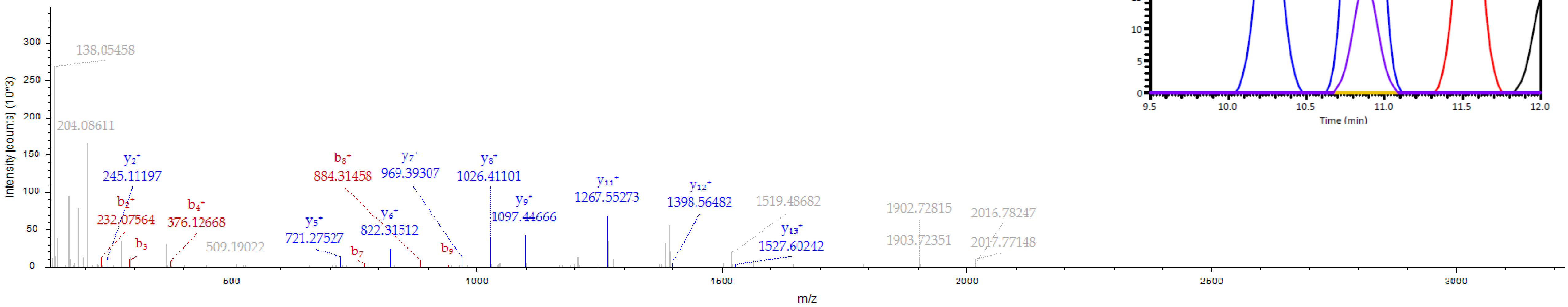

Figure S7. MS/MS spectra for *O*-fucosylated peptides from EGF1-18. N1 was expressed in CHO, U2OS or 3T3 cells with different combination or Fringe, purified from medium, reduced/alkylated, digested with proteases, and the resulting peptides were analyzed by nano-LC-MS/MS as described in Experimental Procedures. Glycopeptides were identified using PMI-Byonic (v.2.10.5) and Proteome Discoverer (v2.1). Spectra for the most representative glycoform of peptides of all conditions from different EGFs are shown, and Extracted Ion Chromatograms (EICs) for each glycoform were generated using Xcalibur Qual Browser (v2.0.3). Peptides are shown from EGF2 (A), EGF3 (B), EGF5 (C), EGF6 (D), EGF8 (E), EGF9 (F), EGF12 (G), EGF16 (H) and EGF18 (I). Since the position of the glycans given by PMI-Byonic and Proteome Discoverer can be erroneous (EGF2, 8 and 9), their position in a monosaccharide form was added to the sequence of each EGFs. The red triangle symbolizes the position of *O*-fucoses and the blue circle the position of *O*-glucoses. Asterisks denote ions that have a similar mass but MS/MS fragmentation does not match the indicated peptide. Data can be found in Supporting Information Tables S4 to S53.
